# Notable predominant morphology of the smallest most abundant protozoa of the open ocean revealed by electron microscopy

**DOI:** 10.1093/plankt/fbac031

**Published:** 2022-07-07

**Authors:** Nina A Kamennaya, Gabrielle Kennaway, Michael A Sleigh, Mikhail V Zubkov

**Affiliations:** French Associates Institute for Agriculture and Biotechnology of Drylands, The Jacob Blaustein Institutes for Desert Research, Ben-Gurion University of the Negev, Campus Sede Boqer, Be'er Sheva 8499000, Israel; Imaging and Analysis Centre, Core Research Laboratories, Natural History Museum, Cromwell Road, London SW7 5BD, UK; Canada Road, West Wellow, Romsey SO51 6DD, Hampshire, UK; Scottish Association for Marine Science, University of THE Highlands and Islands, Oban, Argyll PA37 1QA, UK

**Keywords:** marine heterotrophic flagellates, heterokonts, choanoflagellates, Pacific and Atlantic Ocean, flow cytometry, high-speed flow sorting, scanning and transmission electron microscopy, reverse gravity filtration

## Abstract

In the microbe-driven ecosystems of the open ocean, the small heterotrophic flagellates (sHF) are the chief microbial predators and recyclers of essential nutrients to phototrophic microbes. Even with intensive molecular phylogenetic studies of the sHF, the origins of their feeding success remain obscure because of limited understanding of their morphological adaptations to feeding. Here, we examined the sHF morphologies in the largest, most oligotrophic South Pacific and Atlantic (sub)tropical gyres and adjacent mesotrophic waters. On four research cruises, the sHF cells were flow cytometrically sorted from bacterioplankton and phytoplankton for electron microscopy. The sorted sHF comprised chiefly heterokont (HK) biflagellates and unikont choanoflagellates numerically at around 10-to-1 ratio. Of the four differentiated morphological types of HK omnipresent in the open ocean, the short-tinsel heterokont (stHK), whose tinsel flagellum is too short to propagate a complete wave, is predominant and a likely candidate to be the most abundant predator on Earth. Modeling shows that the described stHK propulsion is effective in feeding on bacterioplankton cells at low concentrations; however, owing to general prey scarcity in the oligotrophic ocean, selective feeding is unsustainable and omnivory is equally obligatory for the seven examined sHF types irrespective of their mode of propulsion.

## INTRODUCTION

In oceanic food webs, the heterotrophic flagellates (HF) of nanoplanktonic size (2–20 μm; Sieburth *et al.*, 1978) play a key role in the microbial loop (Azam *et al.*, 1983). In brief, HF feed on bacterioplankton of picoplanktonic size (0.2–2.0 μm), and in doing so, HF re-mineralize nutrients locked in bacterioplankton biomass, making these nutrients available to phytoplankton: chiefly cyanobacteria and unicellular eukaryotic algae. Historically, the abundance, diversity and biomass of planktonic HF were initially assessed by light microscopy of live cells and then by epifluorescence microscopy of fixed cells (Caron, 1983; Sherr and Sherr, 1983). Sampled plankton, preserved in a defined volume of seawater, were collected on a filter, stained with a fluorescent dye and HF were discriminated from bacteria and pigmented protists by relative size, morphology and lack of photosynthetic pigments. However, accurate sizing, morphological characterization and even registering HF flagella were restricted by the limited resolution of visible light photon-based microscopy (≥0.4 μm). Compared to microscopy, flow cytometric enumeration of HF was much faster and more reproducible (Zubkov *et al.*, 2007), yet HF sizing and characterization using flow cytometry remained problematic. A flow cytometric population of HF cells is usually diverse and its composition requires further characterization.

Morphology-based taxonomic characterization of HF by light microscopy was found to be inadequate and was improved using a combination of light and electron microscopy of cells in concentrated nanoplankton samples and derived enrichment cultures (Sieburth 1978; Sieburth *et al.*, 1978; Cynar *et al.*, 1985; Patterson *et al.*, 1993; Vørs *et al.*, 1995). For example, choanoflagellates [Choanoflagellatea (Cho)] have a characteristic collar termed a choana. The aloricate choanoflagellates (aCho; Craspedida) have only an organic cell coating, while loricate choanoflagellates (lCho), in addition to the organic coating, carry an array of siliceous costal strips organized into a rigid basket termed a lorica that loosely encloses a cell (Leadbeater, 2015). Siliceous baskets of lCho allow identification of specimens to species level (Nitsche *et al.*, 2017). Hence, lists of reported HF species were biased to morphologically identifiable cells, e.g. choanoflagellates, dinoflagellates, *Leucocryptos* and *Telonema*, while smaller cells with only minor distinctive morphological features represented the unidentifiable HF majority (Vørs *et al.*, 1995).

Owing to the limited taxonomically informative morphological features, the *in situ* diversity, evolutionary relatedness and biogeography of HF remained obscure until the introduction of molecular approaches, which transformed the field. Since then, HF have been thoroughly researched mostly through their molecular signatures, genomic content and omics profiles (Pace, 1997; Massana *et al.*, 2006; Thaler and Lovejoy, 2015). Taxon-specific enumeration of HF, initially achieved using fluorescent *in situ* hybridization (Rice *et al.*, 1997), is now commonly inferred from the relative abundance of DNA tags (e.g. 18S rRNA gene fragment) obtained from targeted PCR amplification or random high-throughput sequencing (De Vargas *et al.*, 2015). According to these analyses, oceanic HF are phylogenetically highly diverse with up to 35% domination by stramenopile lineages (Massana *et al.*, 2006). Recent single-cell genomics (Seeleuthner *et al.*, 2018) offered hypotheses for functional diversity of oceanic stramenopiles, e.g. reduced cell motion, trophic stages, capacity to degrade specific polymers, feeding selectivity and gene acquisition from prokaryotes via horizontal transfer. Testing such hypotheses requires studies of HF morphology, locomotion and feeding, because the HF cells are morphologically complex organisms, whose living in the open ocean depends on intercepting sparse bacterioplankton cells and other potential food particles.

Electron microscopy of the HF cells suggests that there are several different methods of movement and food capture in use by different classes of HF. It has long been known that the pattern and direction of water currents created by flagella of HF varies with the beat shape of flagellar undulations and with the presence or absence of stiff lateral hairs known as mastigonemes; these are also related to their methods of capturing food particles (Sleigh, 1964, 1991). When base-to-tip waves pass along a flagellum without stiff hairs, the surrounding water is propelled away from the cell bearing the flagellum, the flagellar base pushes against the cell and the flagellum is known as a pulsellum. By contrast, when planar waves are propagated from base to tip of a flagellum that bears two rows of mastigonemes (tripartite tubular hairs) in the plane of the undulations on a so-called tinsel flagellum, water is propelled toward the cell bearing the flagellum, the base of the flagellum pulls on the cell and the flagellum is known as a tractellum. These two patterns of movement of flagella require that the flagellum is long enough to accommodate a large part of a full wave within its length: we observed that many small HF have flagella that are too short for this and their motion and food collection need investigation.

The reversal of water flow around a beating flagellum that bears stiff lateral hairs in the plane of beat was explained by hydrodynamic calculations; these found that the sum of surface coefficients of numerous lateral hairs as they move through the water during the passage of a wave exceeds those of the main flagellar shaft in the opposite direction (Holwill and Sleigh, 1967). In effect, each individual hair behaves like an oar: before a base-to-tip wave arrives the hair is pointing tip-ward, but as the crest of the wave approaches, it swings backward and after the wave has passed it is pointing toward the base, having executed an oar-stroke. One or two fine terminal filaments are usually present at the tip of each mastigoneme; it is not clear whether these filaments play any part in propulsion, so they are normally neglected.

If the cell to which the flagellum is anchored is free to move, a cell propelled by a pulsellum will move cell-forward, while a cell propelled by a tractellum will move flagellum-forward. Hence, morphologies of HF cells and flagella can inform about the HF propulsion and constrain propulsion models. In their turn, the propulsion models (Higdon, 1979; Nielsen and Kiørboe, 2021) can be used to assess prey interception and consequential growth of different HF cells at a range of prey concentrations and sizes to explain numerical dominance of a certain HF type in the open ocean. Hence, cues for numerical domination, i.e. ecological success, of an HF type or types are concealed in their morphology.

The aim of the present study is to reveal the main HF morphologies in the open ocean. We chose electron microscopy for the morphological examination of HF because it guarantees the required resolution (Hoepffner and Haas, 1990). However, owing to differential cell damage and unquantifiable cell loss during normal sample preparation, electron microscopy is deemed unsuitable for taxon-specific cell enumeration. Because flow cytometry is a robust method for accurate HF enumeration (Zubkov *et al.*, 2007) and purity sorting (Kamennaya *et al.*, 2018; Zubkov and Tarran, 2008), here, we coupled these two techniques to focus on a flow cytometric population of the most abundant, small heterotrophic flagellates (sHF). The sHF cells were separated from cells of bacterioplankton and pigmented protists using flow cytometric sorting at sea and were examined by scanning as well as transmission electron microscopy (SEM and TEM, respectively) ashore in a search for specific morphologies of sHF living in the oligotrophic ocean—the vastest biome on Earth, where the microbial loop role of sHF is particularly significant. The global oligotrophic ocean comprises five (sub)tropical gyres of which the two largest and most oligotrophic, the South Pacific gyre and the South Atlantic gyre, were sampled. By comparing the sHF morphologies which dominate the South Pacific and South Atlantic oligotrophic waters and the adjacent South Pacific and Atlantic mesotrophic waters, we assessed region-related morphological differences using statistical methods. Based on the EM-derived morphometric measurements of the sHF cells and flagella, we classified the seven dominant types and modeled their propulsion and prey interception. The model results suggest a morphological basis for the overall dominance by heterokonts (HK).

## MATERIAL AND METHODS

### Sample collection and processing

**Fig. 1:**
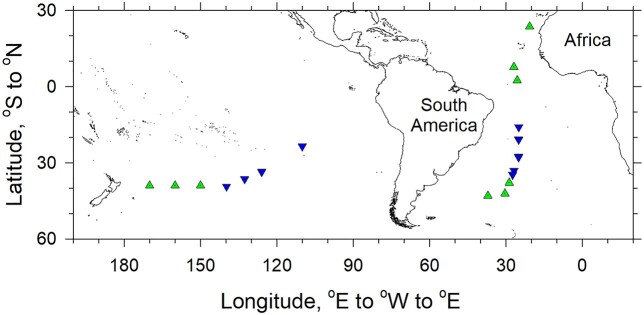
A map of the South Pacific and Atlantic Oceans showing the stations at which the sHF were flow-sorted. Green triangles up and blue triangles down indicate locations in the mesotrophic and oligotrophic waters, respectively.

The main part of the field study was carried out on board the Research Vessel (RV) SONNE during the cruise SO245 (“UltraPac” expedition) to the South Pacific Ocean on January 2016 (Fig. 1, Supplementary Table 1). Auxiliary physical characteristics of the water column (Supplementary Fig. 1a and b) were determined using a conductivity, temperature and depth (CTD) profiler (SBE 911plus, Sea-Bird Scientific). Chlorophyll concentrations were estimated using a CTD-mounted WET Labs ECO-AFL/FL fluorometer (Supplementary Fig. 1c). Seawater was collected using a rosette of 24 20-L Niskin bottles mounted on the frame together with the CTD profiler. For cell enumeration in the photic layer, seawater samples from >10 depths between the surface and 300 m were collected and processed within 30 min of collection. Samples were fixed with 1% (w/v, final concentration) cold paraformaldehyde (PFA, 20% w/v dissolved in seawater and filtered through a 0.1-μm pore size filter), stained with SYBR Green I DNA dye (Marie *et al.*, 1997) and analyzed by flow cytometry (FACSort, Becton Dickinson, Oxford, UK) using the CellQuest software to determine concentrations of total bacterioplankton (Bpl: Bacteria and Archaea), the populations of *Synechococcus* spp. (*Syn*) and *Prochlorococcus* spp. (*Pro*) cyanobacteria (Zubkov *et al.*, 1998) as well as the population of non-pigmented, low nucleic acid-containing (LNA) bacteria plus the small pigmented protists and sHF (Zubkov *et al.*, 2007), respectively (Supplementary Table 2).

To study sHF morphology using SEM and TEM, seawater samples were collected at eight stations (Supplementary Table 1) from one depth in conjunction with bacterioplankton production measurements using GO-FLO sampling bottles. The sampling depth targeted either the deep chlorophyll maximum (DCM) or maximal concentrations of bacterioplankton and sHF when known (guided by the related CTD deployment or microbial counts of samples collected at the same station on the previous day, Supplementary Figs 1c and2c). Because ambient sHF concentrations were low (Supplementary Table 2, <1.5 × 10^6^ cells L^−1^), their direct flow sorting was impractical. Therefore, sHF were concentrated by gentle reverse-filtration (Zubkov *et al.*, 1992). Gravity reverse filtration of live samples through a 1-μm pore size polycarbonate filter concentrated sHF and pigmented protists 25–30×, while concentrations of bacterioplankton remained close to ambient.

The concentrated samples were fixed with ice-cold PFA to 4% (w/v) final concentration, stained with 0.1 μg mL^−1^ Hoechst 33342 (final concentration) and kept on ice until sorted with the custom-configured MoFlo XDP instrument (Beckman-Coulter, High Wycombe, UK) using the Summit 5.4 software (Kamennaya *et al.*, 2018). Sorting purity and recovery of the instrument were controlled using blue (350/440 nm) 1.0-μm beads (Life Technologies) and were cross-checked using EM. Although sHF comprised <1% of analyzed microbial cells, sHF sorting purity was >98%. Occasional bacterial cells were either associated with sHF or in clusters associated with detrital particles, i.e. were genuine sorting targets.

The studies of sHF morphology in the Atlantic Ocean were carried out on board the Royal Research Ships (RRSs) *James Clark Ross*, *James Cook* and *Discovery IV* during the cruises JR303, JC142 and DY084 in October 2014, December 2016 and October 2017, respectively (Fig. 1, Supplementary Table 1). Seawater samples were collected by a rosette of 20-L Niskin bottles mounted on the frame with the CTD profiler in equatorial, (sub)tropical and temperate waters from either the upper mixed layer undisturbed by ship movement (20–25 m) or ~5 m above the DCM. Collected samples were processed as described above for flow cytometry of the same microbial groups (Supplementary Table 2).

In addition, three isolates of *Pteridomonas danica*, a silicoflagellate (Adl *et al.*, 2019), were used to compare variances of the determined cell dimensions of clonal cell populations versus oceanic sHF morphotypes. The *P. danica* cultures of the three isolates were sufficiently dense for flow sorting without pre-concentration. Purity of flow sorting *P. danica* cells from their bacterial prey was >99% according to SEM imaging.

### Microscopy

For SEM analyses, 0.5–4.0 × 10^3^ sHF cells were flow-sorted (Supplementary Table 3) either directly onto 13-mm polycarbonate filters (pore size: 0.2 μm) under low vacuum during the Pacific cruise or into 5-mL polypropylene tubes, the content of which was filtered onto 13-mm polycarbonate filters. The cells deposited onto the filter were dehydrated in an ethanol series and critical point dried using 99.9% hexamethyldisilazane (Sigma-Aldrich). Sample dehydration was performed using low-vacuum filtration that prevented loss of sorted cells. The dehydrated filters were stored in a desiccator at room temperature. Prior to SEM analyses, the filters were sputtered with Au/Pd (3:2) to a thickness of 10 nm using a Cressington High-Resolution Sputter Coater coupled with an MTM20 film thickness controller (Cressington Scientific Instruments, Watford, UK). SEM imaging was carried out using a ZEISS Ultra Plus field emission SEM (Carl Zeiss microscopy UK, Cambridge, UK) at the Imaging and Analysis Centre of the Natural History Museum in London, UK.

To locate clusters of sorted cells, a low-resolution scan of whole filters was first performed using an automated motorized stage, coupled with a ZEISS ATLAS image capture system with the SEM operating at 5 keV using the secondary electron detector. Cell clusters and single cells were imaged at high resolution at 5 keV using the low resolution images as navigational aids. Within each located cluster, at least 8%, but usually >15% of total sorted sHF cells were counted using lower-resolution SEM (Supplementary Table 3). All sHF cells imaged at high resolution were measured: 497 and 192 sHF cells in the samples collected in the Pacific and Atlantic Ocean, respectively (Supplementary Table 4). The sHF cells imaged at intermediate resolution (fields of view: from 56 × 37 to 246 × 161 μm) were counted to assess the relative abundance of different cell types (Supplementary Table 5).

For TEM analyses, 0.5–5.0 × 10^3^ sHF cells were flow-sorted directly on Formvar/carbon–covered 200 mesh copper grids (Agar Scientific, Stansted, UK), stained with 2% w/v gadolinium (aqueous solution), rinsed with pure deionized water and stored in a desiccator for analysis ashore. The grids were examined at 200 keV with a JEOL 2011 LaB6 TEM instrument [JEOL (UK) Ltd, Welwyn Garden City, UK] fitted with a Gatan UltraScan 1000 camera (Gatan UK, Oxford, UK) at the University of Warwick or at 100 keV with a *Hitachi H-7650* TEM instrument fitted with AMT 2 K × 2 K digital camera system at the Bioimaging Laboratory of the Royal Botanic Gardens, Kew, in London, UK.

### Determination of cell and flagellar dimensions

The dimensions were used for differentiating sHF morphotypes, estimating their biovolumes, statistical analyses of their biogeography and modeling their prey interception. Linear cell and flagellar dimensions were determined from both SEM and TEM micrographs and cross-compared, where possible, using standard statistical tests. In addition, linear dimensions of mastigonemes and their terminal filaments were determined from TEM micrographs of higher magnification. Under the assumption that cell shape could be approximated by a prolate sphere, cell volumes were calculated as *V* = π/6(*l* × *s* × *d*); where *l* is the length of the longest cell dimension, *s* is the length at 90^o^ to the longest cell dimension and *d* is the cell thickness that was assumed equal to s. Flagellar length (*l*) and thickness (*s*) were measured for flagellated cells, and the flagellar biovolume was estimated as *V* = π/4(*l* × *s* × *d*), assuming that flagellum shape could be approximated by an oval cylinder (Olenina *et al.*, 2006). For modeling propulsion of sHF, the measured linear cell dimensions were corrected for 50% shrinkage of PFA-fixed cells (Sherr and Sherr, 1993), including flagellar thickness, while flagellar length remained uncorrected. The sHF cell biomass was calculated using the C:unit biovolume ratio of 0.2 pg C μm^3^ (Sherr and Sherr, 1993; Zubkov *et al.*, 2000).

Standard *F*-test and *t*-test were used to compare variance and mean, respectively. To compare dimension variances of different types of sHF and model *P. danica* cells without a bias related to differences in cell sizes, we calculated the relative differences from the mean (difference between direct measurement minus mean divided by the mean) and then compared the variances of the relative differences or relative variances using the *F*-test. This approach also allowed size-unbiased comparison of variances of cell and flagellar dimensions.

## RESULTS AND DISCUSSION

A *Synechococcus* spp. concentration of ~6 × 10^6^ cells L^−1^ (Supplementary Table 2) guided delineation between the oligotrophic and mesotrophic waters (Zubkov *et al.*, 1998) of the open ocean (Fig. 1). The concentration of total bacterioplankton was generally below and above 10^9^ cells L^−1^ in the oligotrophic and mesotrophic waters, respectively. The LNA bacteria, primarily comprised of the SAR11 alphaproteobacterial group (Mary *et al.*, 2006; Gomez-Pereira *et al.*, 2013, Supplementary Fig. 3), numerically dominated bacterioplankton of the oligotrophic waters: they comprised 54.0 ± 6.1% and 54.7 ± 3.1% in the South Pacific and South Atlantic oligotrophic waters, respectively. The percentage of LNA cells in the bacterioplankton of the mesotrophic waters was slightly lower: 47.3 ± 4.0% and 47.0 ± 7.1% in the South Pacific and Atlantic mesotrophic waters, respectively. *Prochlorococcus* spp. cyanobacteria were the most numerous sub-dominant group in the oligotrophic waters; they comprised 15.5 ± 4.2% and 27.7 ± 4.2% of bacterioplankton in the South Pacific and South Atlantic oligotrophic waters, respectively. *Prochloroccocus* spp. concentrations in the mesotrophic waters were more variable, ranging from 2 × 10^6^ cells L^−1^ to blooming 6.2 × 10^8^ cells L^−1^. Concentrations of the small pigmented protists were 2–10× higher in the mesotrophic versus oligotrophic waters, whereas sHF concentrations were more variable but generally 2× higher in the mesotrophic waters.

**Fig. 2:**
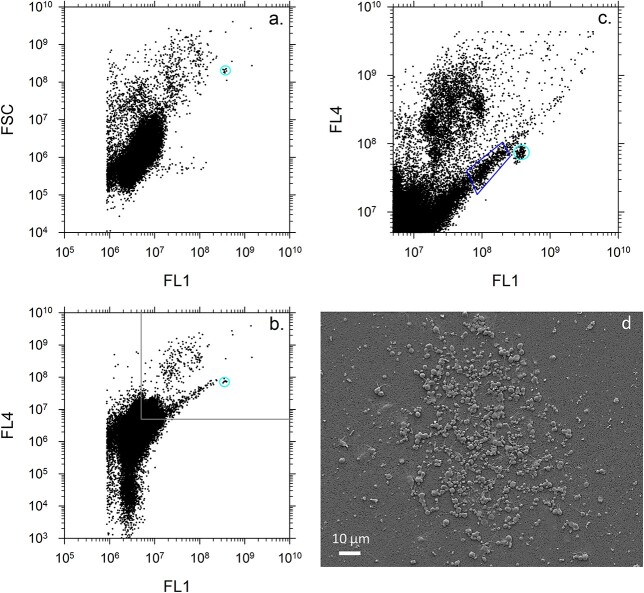
Characteristic flow cytometric signatures of the concentrated, DNA-stained oceanic microbes used for targeted flow sorting of the sHF with their corresponding SEM micrograph. (**a**) A log plot of 10^5^ dots of Hoechst-DNA fluorescence above the set threshold (FL1) excited by the first 355 nm laser versus shallow angle or forward light scatter (FSC) showing the dominating bacterioplankton population and scatter of the small protists. The cyan oval indicates 1.0-μm reference beads. (**b**) A log plot of 10^5^ dots of Hoechst-DNA fluorescence (FL1) versus red fluorescence (FL4) excited by the first laser showing the bacterioplankton and small protists. The cyan oval indicates 1.0-μm reference beads. The dark gray lines indicate the zoomed into area shown on the plot (c). (**c**) A log plot of 2 × 10^6^ dots of Hoechst-DNA fluorescence (FL1) versus red fluorescence (FL4) excited by the first laser showing the bacterioplankton and small protists with the dominant population of sHF, indicated by the blue polygon. Notice the scale change to amplify the targeted population and 1.0-μm reference beads (cyan oval). (**d**) A low-resolution SEM micrograph showing a cluster of the flow-sorted sHF cells.

**Fig. 3:**
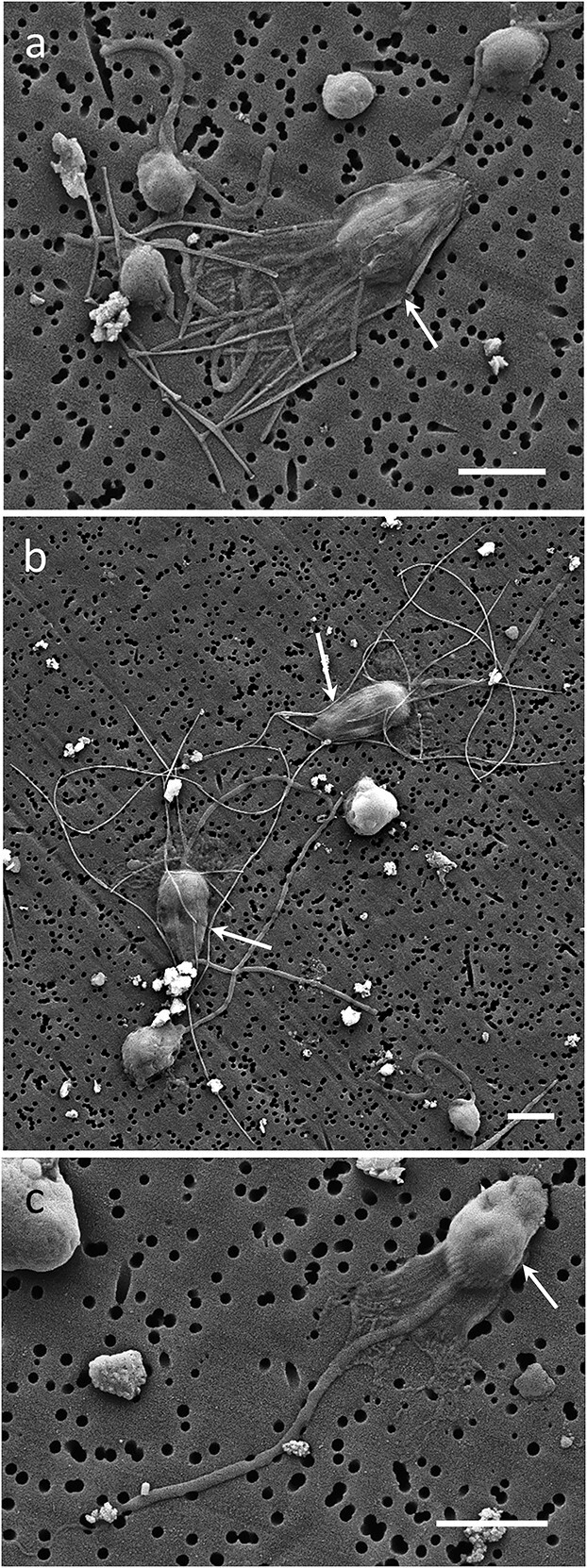
Example SEM micrographs of the examined morphotypes of Cho (white arrows): lCho (a) *S. oceanica* and (b) *C. conicella* and (c) an aCho cell. Scale bar = 2 μm.

**Fig. 4:**
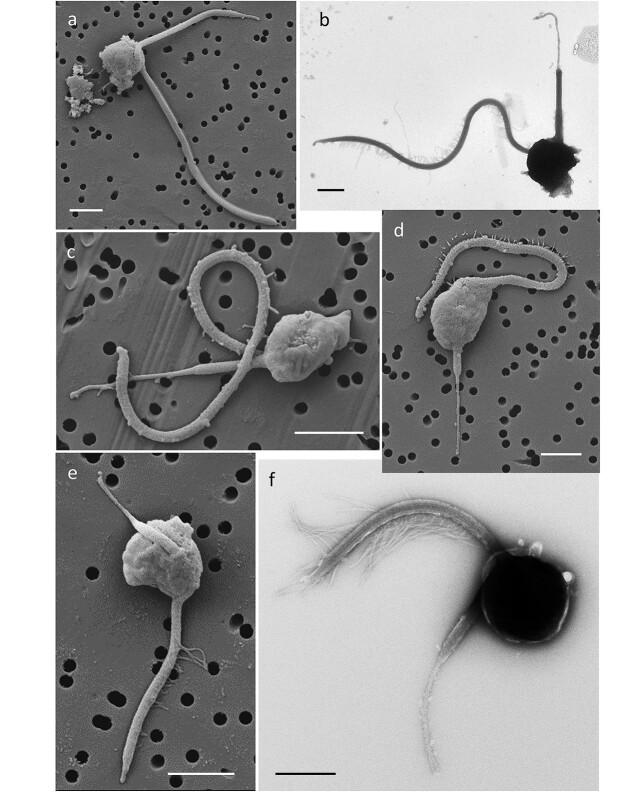
Example SEM (a, c–e) and TEM (b, f) micrographs of the flow-sorted morphotypes of HK: (a, b) the lcHK, (c) the mcHK, (d) the scHK, and (e, f) the stHK. Scale bar = 1 μm.

SEM analyses of the sorted cells from the groups of oceanic microbes and *P. danica* cells confirmed the purity of their sorting; the sorted cells were of the same morphology within each category (Supplementary Figs 3 and 4). By contrast, the sorted sHF cells were morphologically heterogeneous (Figs 2–4, Supplementary Fig. 5). General examination of EM micrographs of the sHF cells flow-sorted from samples collected in the Pacific and Atlantic oceans revealed two ubiquitous, conspicuous morphologies: unikont collared flagellates and HK biflagellates.

The quantitative composition of the sHF was determined at seven stations in the South Pacific Ocean at which the sHF cells were flow-sorted directly onto the filter to form a tight cluster of cells (<300 μm diameter, e.g. Fig. 2d, Supplementary Fig. 4, *P. danica* L, a) for subsequent SEM-based counting, whereas at stations in the Atlantic Ocean, the sorted sHF were first collected in tubes and then filtered. Hence, the sorted cells were distributed evenly over the filtering area, which made their SEM enumeration impractical. However, the numbers of the analyzed cells (Supplementary Table 4), although possibly biased to rarer morphotypes favored by the examiners (e.g. lCho in the South Pacific oligotrophic waters), still indicated the relative abundance of sHF morphotypes, as the comparison with the counted sHF shows (Supplementary Table 4b vs. Supplementary Table 5b).

**Fig. 5:**
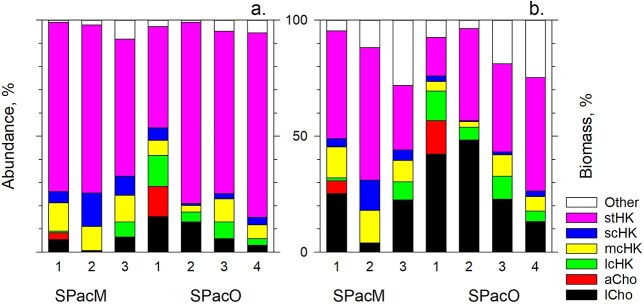
Relative abundance (**a**) and biomass (**b**) of the seven dominant morphotypes plus other cells which comprised the sHF flow-sorted from samples collected in the South Pacific mesotrophic (SPacM) and oligotrophic (SPacO) waters. The morphotypes are the stHK, scHK, mcHK and lcHK and the aCho and lCho, respectively.

The majority (87.0 ± 8.9%) of the sHF cells comprised HK (Fig. 5a, Supplementary Tables 4 and 5) that were 1.5–2.0 μm in diameter (Supplementary Table 6) and bearing two characteristic flagella positioned at an angle of <90^o^ to one another (Fig. 4, Supplementary Fig. 5). The longer tinsel flagellum carried numerous mastigonemes (1–2 μm long tripartite tubular flagellar hairs), apparently in two rows (Fig. 4f, short-tinsel heterokont (stHK); Supplementary Fig. 5b and d), each of which ended with a single 0.3–0.4 μm (e.g. 0.35 ± 0.03 μm of stHK) long terminal filament or bristle (Andersen *et al.*, 1991) visible only by TEM at ≥×3000 magnification. The shorter non-tinsel or “caudal” flagellum commonly narrowed down to form a 0.6–1.7 μm long, whip-like tip (Fig. 4, Supplementary Fig. 5) and lacked mastigonemes. The ascribed HK morphology is characteristic for the Stramenopiles (Adl *et al.*, 2019). This is the first morphological evidence (to our knowledge) of Stramenopiles being so abundant in the open ocean. Neither organic nor mineral scales covered the HK cells (Fig. 4), while the collared unikonts had organic coverings with a lorica of various complexities or without a lorica (Fig. 3).

The second (9.4 ± 9.2%, Fig. 5a and Supplementary Table 5a, b) ubiquitous group was unikont cells with their single flagellum surrounded by a collar of microvilli. Their flagella do not have mastigonemes, in contrast to HK tinsel flagella, but generally have a narrowed tip (whip) section. This morphology defines the taxonomically coherent class of Cho (Leadbeater, 2015). The remaining material was either cell debris associated with sHF, occasional larger *Telonema* or dinoflagellate cells or cells of inconspicuous morphology, generally spheroid shape, which were recorded to complete assessment of relative abundance and biomass of different sHF types (Fig. 5). Cumulatively, those other cells (<5–10 specimens) comprised the minority of sHF (3.6 ± 2.7%) and were sampled insufficiently for inclusion into the morphological study. Because the cells of these rare morphologies were larger, they contributed 5–28% to total biomass, while the HK and choanoflagellate contributions totaled 36–84% and 4–57%, respectively (Fig. 5b and Supplementary Table 5e). The absolute HK and choanoflagellate concentrations varied at 4.49–14.67 × 10^5^ and 0.01–2.34 × 10^5^ cells L^−1^, respectively (Supplementary Table 5c and d); however, owing to the larger size of choanoflagellates, the absolute choanoflagellate and HK biomasses were comparable at 0.217–0.684 and 0.032–0.527 μg C L^−1^.

### Dominant sHF morphotypes

Choanoflagellates were practically omnipresent and were observed at 11 out of the 13 stations sampled in the Atlantic and at all 8 stations sampled in the Pacific Ocean. While we describe their taxonomic diversity elsewhere, here, we present the lCho as an aggregate group together with the group of aCho for comparing sHF morphotype abundance and biomasses (Supplementary Tables 4 and 5). Although we cannot exclude that some of the flow-sorted lCho lost their lorica during sample preparation and, consequently, were identified as aCho, there are several lines of evidence that the majority of the analyzed aCho are genuinely aloricate. First, the abundances of loricate and aCho do not co-vary, the aCho were relatively abundant at two out of seven stations unrelated to abundance of lCho (Supplementary Table 5). Second, the cells of aCho were significantly smaller with a shorter flagellum than the cells of lCho (Supplementary Table 6). Third, there are independent earlier reports of aCho presence in the open ocean (Vørs *et al.*, 1995).

For comparing growth and propulsion of different sHF cells, we selected three choanoflagellate representatives for which we collected sufficient morphometric data (Supplementary Table 6): the two recently described abundant oceanic species of lCho: *Stephanacantha oceanica* (Thomsen *et al.*, 2020) and *Coronoeca conicella* (Thomsen *et al.*, 2021) as well as the aCho (Fig. 3).

HK were observed at all (*n* = 21) stations sampled in both the Atlantic and Pacific Oceans. Furthermore, HK were predominant among the sorted sHF cells (Fig. 5 and Supplementary Tables 4, 5). Compared to lCho (Leadbeater, 2015), the morphology of oceanic HK stramenopiles was not investigated in detail previously (to our knowledge) beyond reports that they were small and hence bacterivorous flagellates with one or two flagella (Massana *et al.*, 2006). The diversity of HK has been previously studied by molecular methods, which provided limited guidance for morphological differences. In the absence of such guidance, we were assisted by flow sorting hundreds of sHF cells, which gave a rare opportunity of examining and measuring numerous (*n* = 502) specimens.

Based on dimensions of HK flagella and cells, as well as their overall morphology, we made out four morphological types omnipresent in both oceans (Supplementary Tables 4 and 5). In brief, the HK with the longest tinsel and caudal (non-tinsel) flagella were named as the long-caudal heterokonts (lcHK, Fig. 4a, b). The HK with medium-size caudal flagella were named the medium-caudal heterokonts (mcHK, Fig. 4c). The HK, which had very short caudal flagella with twice-longer whip (narrowed tip portion) and occasionally only a whip was visible, were named as the short-caudal heterokonts (scHK, Fig. 4d). The HK, which had the shortest tinsel flagella, were named as the stHK (Fig. 4e, f). The stHK were predominant within heterokonts as well as within the sorted sHF cells (Supplementary Tables 4 and 5). The scHK and mcHK were more abundant in the mesotrophic than in the oligotrophic ocean, whereas the lcHK showed the opposite tendency. The TEM images recorded rare cases of the waved tinsel flagella of the lcHK (Fig. 4b and Supplementary Fig. 5) and more numerous cases of half-bended tinsel flagella of the stHK (Fig. 4f). Such flagellar profiles are characteristic of live sHF of this size (personal light microscopy observations of M.A.S.). The TEM images aided modeling of HK motion and prey interception (see below).

Based on the similarity of the HK morphotypes at the ocean-scale (Supplementary statistical analyses, Supplementary Tables 7–11), we pooled together all the acquired morphometric data to assess their mean values for the entire open ocean (Supplementary Table 6). The differences between the mean cell and flagellum sizes are significant except the width of the stHK and scHK cells. The caudal flagellum of the scHK is significantly shorter than the caudal flagella of either the stHK or mcHK. The lcHK have the longest flagella. The whip of the caudal flagellum is the most variable dimension in all HK. Even so, the whip of the stHK is half the length of the whips of the other three HK morphotypes.

To assess how the size range of the oceanic sHF relates to the variance of their dimensions, we compared the sHF variances with the size variances of the three isolates of *P. danica* (Supplementary Fig. 4). The *P. danica* isolates and the sHF were within the same size range (Supplementary Table 6). To reduce the ocean-scale variance, we selected the population of the stHK, which were sampled at the SPacO_3 station (Supplementary Tables 1 and 6) to make comparisons of means and particularly variances with the clonal isolates of *P. danica* fairer. Even the smallest *P. danica* cells were significantly bigger with a longer tractellum than the stHK cells. It was notable that the sizes of three isolates of *P. danica* were significantly different from each other. Although the differences between the small- and medium-size isolates of *P. danica* were marginal, the large-size isolate of *P. danica* was >50% bigger with a >20% longer flagellum, providing a scale of size variance between isolates of the same species, which was comparable to the differences in sizes within each of the three choanoflagellate and the four HK types, apart from the tinsel flagellum of the stHK.

Although size variance cannot predict a rate of growth, high size variance indicates a growing population, i.e. in a growing population, there are more cells at different stages of cell growth, the range of cell sizes is wider and, consequently, size variance is higher. Variance could also be used as an index to compare the level of conservation of the flagellar length of different species or morphotypes. However, because variance is a function of size, variance of smaller cells is generally lower (Supplementary Table 6). Therefore, to negate the size differences, we also compared relative variances (Supplementary Table 12). The relative variances of both cell and flagellar sizes of the complex group of lCho, comprised of several species, was the highest as expected. The relative variances of the *P. danica* isolates were the lowest because their cells were flow-sorted at the stationary phase of population growth, and hence their relative variance of 0.081–0.097 should indicate the minimal population growth. Unsurprisingly, the relative variance of cell sizes of the stHK population growing in the ocean was higher: 0.151–0.153. The relative variance of the short tinsel flagellum length was, however, similar to the relative variance of flagellum lengths of the three isolates of *P. danica*, i.e. similarly conserved. The relative variance of the cell sizes of the stHK was similar to the relative variance of cell sizes of lCho species, *C. conicella* and *S. oceanica*. It is noteworthy that, unlike the stHK, the relative variance of the flagellum length of the *C. conicella*, *S. oceanica* and aCho group is as high as relative variances of their cell dimensions, suggesting that the pulsellum length is less conserved than the tractellum length, particularly of the stHK and scHK, perhaps, owing to the nature of their propulsion.

### Comparing feeding motions of the HK and choanoflagellates

To explain the numerical dominance of oceanic sHF by the HK, in general, and the stHK, in particular, we compared feeding motions of the seven common sHF morphotypes: the four HK versus the three choanoflagellates. In the absence of direct measurements, we estimated doubling times of oceanic sHF using the results of feeding experiments with the sHF proxy model, the large-size *P. danica* isolate (*P. danica* L, Zubkov and Sleigh, 2005) as explained below.

**Fig. 6:**
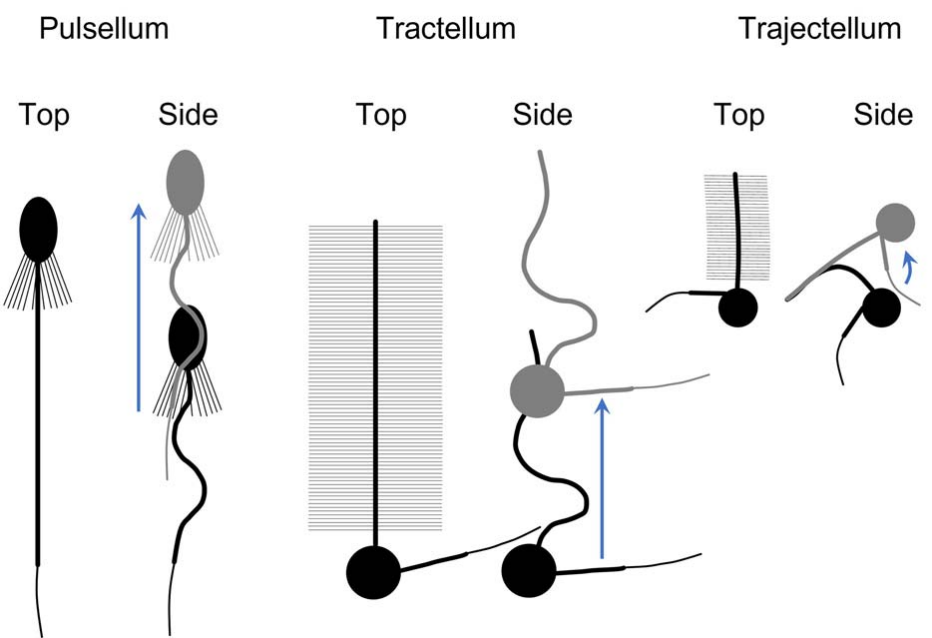
Diagrams to illustrate the pulsellum, tractellum and trajectellum motion. The top view shows the full length of the propelling flagellum including mastigonemes. The side view shows the propelling flagellum wave or bending. The black image shows the initial cell position and the gray superimposed image shows the final cell position after the stroke. The blue arrows indicate the direction of locomotion.

Synthesis of the acquired morphological data sufficed to constrain three basic (geometric) propulsion models based on a common concept of a cell cross-section moving through water by flagellar beating. Because the beating flagellum propagates a wave, a part of the flagellum is engaged in bending to get a purchase on water. Consequently, the wavelength is always shorter than the flagellar length. Owing to the shortness of their flagella, i.e. <5× cell diameters (Higdon, 1979), these seven sHF cannot create much more than one complete wave and the wave length is the maximal distance of cell propulsion by one wave in idealized conditions, i.e. 100% efficiency of the propulsive motion of water by the flagellum and 0% loss of the cell motion owing to drag. Hence, we assumed wavelength-long cell propulsion by flagellar undulation for the pulsellum (*S. oceanica*, *C. conicella* and aCho) and tractellum (*P. danica* L, lcHK, mcHK and scHK) models (Fig. 6). Propulsion of the stHK (Fig. 4e,f), whose tinsel flagellum is too short to create even a complete wave, was described by the proposed trajectory or trajectellum (to harmonize terms) model (Fig. 6), as explained below.

In the following three models, we have described motion in terms of the flagellar beating propelling the cell through the water rather than propelling water past the cell. It is appreciated that the actual locomotion of the cell depends on the propulsive activity of the flagellum and the opposing drag that is dependent on the size of the cell body and the presence of other cell structures such as the lorica of lCho. What is important in the feeding of the cells is the rate at which water carrying food particles is moved over food-collecting surfaces. Here, we call this water flow over feeding surfaces propulsion irrespective of the amount of locomotion of the cell (Fig. 6) because the amount of drag varies. Furthermore, the trajectellum of the smallest cells produces a complex motion with little actual progression (Fig. 6).

Just as viscous forces play a major role in the propulsion of water by flagella (e.g. Higdon, 1979; Holwill and Sleigh, 1967), these forces must also be expected to play a role in the interception and retention of food particles. However, particle capture at the Reynolds numbers involved has not (to our knowledge) been studied by experts in fluid dynamics. The extent to which viscous forces increase the effective dimensions of both the cell and its organelles under working conditions is unknown. Therefore, in the following discussion, we have assumed that it is negligible, or at least that viscous forces affect all types to a similar extent.

### The tractellum feeding motion

**Fig. 7:**
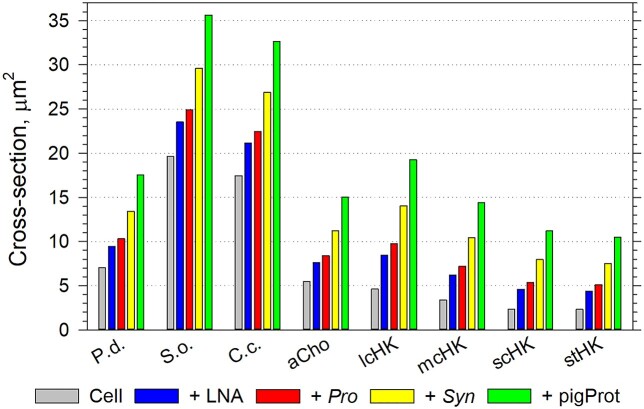
Comparison of the dependence of prey interception cross section of the *P. danica* isolate (*P.d.*) of large cells, the representative choanoflagellate species, *S. oceanica* (*S.o.*), *C. conicella* (*C.c.*) and average aCho, as well as the lcHK, the mcHK, the scHK and the stHK in the open ocean on the size of the prey, when presented with no prey (Cell), LNA containing bacterioplankton (LNA), *Prochlorococcus* spp. (*Pro*), *Synechococcus* spp. (*Syn*) and pigmented protist (pigProt) prey. The dotted horizontal guidelines aid comparisons.

While the tractellum beats (Fig. 6), the HK cell intercepts and captures prey across the area comprised of the cross-sections of the cell and its whipped caudal flagellum (Supplementary Fig. 7a). Because interception depends on the size of both predator and prey, the prey diameter should be added to the predator cell diameter, its caudal flagellum width and the whip width to calculate the prey interception cross-section (Fig. 7). In estimating the tractellum wavelength, we were assisted by rare TEM images, which captured the stroke of the lcHK (Fig. 4b and Supplementary Fig. 5), the wavelength of which was ~47% of the tractellum length; such images resemble those seen by stroboscopic light microscopy (Sleigh, 1964). As already mentioned, we assumed that the stroke wavelength equals the distance that the cell is propelled by a single beat cycle (Fig. 6). Consequently, during a stroke, the lcHK cell intercepts prey from a prey interception volume calculated by multiplying the prey interception cross-section by the tractellum wavelength (Fig. 7 and Supplementary Fig. 7a). The prey interception volume during a single stroke of the mcHK and scHK cells was calculated analogously by multiplying their prey interception cross-sections by their respective wavelengths or stroke motions.

It was more challenging to determine the wavelength of the mcHK and scHK cells because their presumably more flexible tractella were generally found wrapped round their cells (Fig. 4c, d and Supplementary Fig. 5) often in a characteristic shape of a figure of eight. In the absence of direct observations, we derived the wavelength of the mcHK and scHK cells from the lcHK wavelength by correcting for the differences in their cell cross-section (including the caudal flagellum) relative to the “working area” of their tractella (Fig. 6). The flagellar working area was estimated by multiplying the tractellum length by twice the length of their mastigonemes (1.71 μm of scHK and mcHK, 1.93 μm of lcHK) because of the mastigoneme arrangement in two opposing tightly spaced (~0.1 μm) rows. Because only the top ~85% of the tractellum length bore mastigonemes, the working area was corrected accordingly. The estimated wavelengths of the mcHK and scHK cells were 52% (close to the lcHK) and 68.5% of their tractellum lengths, respectively. The 68.5% estimate suggests that the scHK cell propulsion is close to the maximum achievable by the sine wave of 45^o^ (Higdon, 1979), i.e. 72% at ~5.7 μm stroke^−1^ (Supplementary Fig. 7a). The estimated stroke motions of the lcHK and mcHK were similar ~4.7 μm stroke^−1^, 1 μm shorter than of the scHK.

### The pulsellum feeding motion

In the case of choanoflagellates, a pulsellum stroke pushes the cell through the water (Fig. 6), while the cross-section of the cell is considerably enlarged (compared to the HK cross-sections) by the protruding collar cone (Fig. 3 and Supplementary Fig. 7a). Because, unfortunately, no waved flagella were found preserved on TEM images, the pulsellum wavelength was deduced indirectly. We assumed that the pulsellum of a free-swimming choanoflagellate produces a sine wave, as earlier observations (Sleigh, 1964) and theoretical studies (Higdon, 1979) suggest. We assumed that, when the cell diameter equals the height of the sine wave, the sine wave with an optimal pitch angle of 45^o^ (Higdon, 1979) would push a cell the full wavelength, i.e. 72% of the pulsellum length. The protruding collar slows cell propulsion proportionally to the ratio of the maximal cross-section of the collar relative to the cross-section of the cell. The apical whip was not included in calculations because its role in propulsion (although likely) has not been established. Whereas the collar of the aCho merely increased the cross-section by a factor of 2.0×, the collar of *S. oceanica* and *C. conicella* increased the cross-section 4.5× and 4.4×, respectively (Supplementary Fig. 7a). Consequently, their wavelengths or stroke motions were at 2.6, 1.8, 1.6 μm stroke^−1^, respectively. Perhaps, because it works in the opposite way, the pulsellum stroke seemed less “efficient” than the tractellum stroke of HK. To calculate the prey interception cross-section, the prey diameter was added to the maximal collar diameter (Fig. 7). The single-stroke prey interception volumes were calculated by multiplying the interception cross-section by the respective stroke motion (Supplementary Fig. 7a).

Although it was proposed that the lorica could stabilize cell movement and presumably increase prey capture efficiency by creating drag (Asadzadeh *et al.*, 2019), we decided to refrain from direct accounting for the lorical cross-section in our calculations to reduce complexity. Furthermore, rather than increasing lorical drag, oceanic choanoflagellates seem to build their lorica to minimize that drag, e.g. *S. oceanica* cells are enclosed in a streamlined cone of petal-form costa (Fig. 3a). Instead, we considered that the lorica forms scaffolding for holding the collar as a cone protruding from the cell and, hence, increases cross-section up to 4.5× of *S. oceanica* for intercepting prey compared to 2.0× of aCho (Supplementary Fig. 7a).

### The trajectellum feeding motion

The trajectellum model was most challenging to constrain because it reconstructed an unusual motion of the stHK cell created by its tinsel flagellum (Fig. 6), whose length is too short (only 2.2× cell lengths, Fig. 4e, f, Supplementary Fig. 5 and Supplementary Table 6) to propagate even a single complete wave. A cue to how the stHK could move was deduced from the ratio between the cross-section of the cell plus whipped caudal flagellum and the mastigoneme-covered working area (85%) of the tinsel flagellum with the mean mastigoneme length of 0.99 ± 0.19 μm (Fig. 4f and Supplementary Fig. 5). Apparently, the ratio indicates that the purchase of the ~40% top part of the tinsel flagellum sufficed to outweigh resistance of the cell cross-section drag (Fig. 6). In other words, when the tinsel flagellum unbends, the cell rather than the flagellar top would move. Most of the stHK cells observed by TEM (Fig. 4f) had bent tinsel flagella, suggesting that this is the starting (relaxed by cell fixation) position of a stroke. The tinsel flagellar bending would engage mastigonemes in water purchase (Fig. 6). When the tinsel flagellum straightens, the cell starts moving forward, “disengaging” mastigonemes toward the tinsel root. The angle of the bent tinsel flagellum suggests that a stroke tracks the cell by ~90^o^. Consequently, the cell moves along an elliptic trajectory (calculated using Ramanujan’s approximation 3, Ramanujan, 1914) with the pivotal point at ~40% of the tinsel length from the top. The starting elliptic radius would be the radius of a circle with a perimeter equivalent to 4× lengths (because of 90^o^ bending) of the 60% tinsel (the bent tinsel part) plus the cellular radius. The final elliptic radius equates to the straightened 60% tinsel length plus the cellular radius. Although such trajectellum propulsion looks cumbersome (Fig. 6), it is actually rather efficient because, in fact, a tinsel flagellum that is merely 3.4 μm long (Supplementary Fig. 6b) could propel a stHK cell (~1.6 μm diameter, Supplementary Fig. 6a) a whole 3.9 μm (Supplementary Fig. 7a). In contrast to the smooth motion achieved by a pulsellum or tractellum, a sequence of straightening strokes of a trajectellum will propel the cell in a series of jerks (Fig. 6). As a result of this erratic motion, there is a constantly changing flow of particle-laden water over the food capturing cell surface. Compared to propulsion by a tractellum or pulsellum that by a trajectellum is doubtless inferior in keeping direction; however, in the boundless oceanic waters with randomly dispersed food particles, this seems as little or no handicap.

### Assessing rates of flagellar beating

Whereas morphological observations, assisted by the geometric models, could deduce the sHF cell motion during a single stroke (Fig. 6), reconstruction of overall cell motion requires an additional key parameter, i.e. the flagellar beating or stroke frequency. Measuring the frequency needs live detailed high-resolution observations of sHF movement (Sleigh, 1989), which is difficult enough to do ashore and impractical to do at sea. After considering how closely the assumed cell motion corresponds to live observations, we can then go on to consider whether the motion and food capture mechanisms described enable the various sHF types to survive at the observed ambient food particle concentrations.

In the absence of direct observations, we estimated stroke frequencies of the oceanic sHF indirectly using the large-size *P. danica* as a proxy model organism of the sHF. For the *P. danica* isolate, we have the required morphological measurements (Supplementary Table 6) and the experimental data of its feeding on model bacterial prey (Zubkov and Sleigh, 2005) to determine *P. danica* doubling time as well as the stroke frequency (Supplementary Fig. 6b) and stroke motion (Supplementary Fig. 7a). The cell size (3.1 μm diameter) of the *P. danica* isolate is close to the size of *S. oceanica* and comparable with the other sHF (Supplementary Table 6). The model prey size and initial concentration (0.57 μm, 2.2 × 10^9^ cells L^−1^, respectively) simulated the maximal size and concentration (0.6 μm, 2.1 × 10^9^ cells L^−1^) of oceanic bacterioplankton (Supplementary Table 2 and Supplementary Fig. 2a), which is essential for the realistic assessment of growth (Fenchel, 1982a, 1982b). In our experiments (Zubkov and Sleigh, 2005), the free-swimming *P. danica* intercepted >6 prey cells h^−1^, assimilated prey with 20% efficiency and would divide every 5.4 days, if the prey remains at the initial concentration of 2.2 × 10^9^ cells L^−1^ (Supplementary Fig. 7b).

Transposing this doubling time of *P. danica* to oceanic sHF should, however, be done with a certain caution because *P. danica* is living in coastal rather than oceanic seawaters, and mature *P. danica* cells prefer to feed attached to particles (Christensen-Dalsgaard and Fenchel, 2003). In our feeding experiments (Zubkov and Sleigh, 2005), large cells of *P. danica* were “forced” to feed free-swimming by using stationary phase prey-depleted “swarmer” cells in the absence of particles for attachment. Therefore, the doubling time of 5.4 days at 2.2 × 10^9^ prey cells L^−1^ should be viewed as an upper estimate.

There is a certain similarity between the sHF cells (which use their cell cross-section to intercept prey) and the osmotrophic prokaryotes (which take up dissolved nutrients across the cell surface). The nutrient uptake of osmotrophs and hence their doubling time is proportional to the ratio of cell volume-to-surface (Kendall, 1923), i.e. the linear size of a cell—in other words, the smaller the cell, the more often it divides. By analogy, the sHF doubling time should be proportional to the ratio of cell volume-to-cross-section, which is also the linear size. To normalize the cell shape differences of the sHF types, their corrected linear sizes were computed as the diameter of a sphere whose volume equates to the total cellular volume, including flagella and collar. By proportioning the doubling time of the large-size *P. danica* to the corrected linear sizes of the sHF, we can, hence, estimate the doubling times of their types from 5.4 days for *S. oceanica* to 2.8 days for the stHK (Supplementary Fig. 7b). Using the derived doubling times and assuming that the sHF prey capture efficiency is 100% (for simplicity) and prey assimilation efficiency of oceanic sHF and *P. danica* are similar at 20%, we can estimate the number of intercepted model prey cells required for doubling: from 108 cells for the stHK to 825 cells for *S. oceanica*. To achieve their respective doubling times, they need to intercept a model prey cell every 37 and 9.5 minutes, respectively. At the model prey concentration of 2.2 × 10^9^ cells L^−1^, the stHK and *S. oceanica* would need to intercept all bacteria from 211 and 817 μm^3^ every second using their model prey-specific interception volumes of 19 and 45 μm^3^ stroke^−1^ by doing 11 and 18 strokes s^−1^ or Hz, respectively (Supplementary Fig. 6b).

The deduced tractellum frequencies of the lcHK, mcHK and scHK were similar (9.0, 9.5 and 7.9 Hz, respectively) irrespective of the tractellum length (Supplementary Fig. 6b). The twice higher pulsellum frequencies of *S. oceanica*, *C. conicella* and aCho (18.2, 20.8 and 21.0 Hz, respectively) were also similar among the choanoflagellates irrespective of the pulsellum length. These values seem realistic because stroke frequencies of >20 s^−1^ were recorded for longer flagella (Sleigh, 1989) and comparable stroke frequencies of the large-size *P. danica* cells were determined experimentally (Zubkov and Sleigh, 2005). Apparently, the *P. danica* stroke frequency was lower and more variable at 17 ± 5 Hz with higher prey concentrations >3 × 10^9^ model prey cells L^−1^ and were higher and less variable at 29 ± 2 Hz with prey concentrations <2.2 × 10^9^ cells L^−1^, with a maximum of 31.5 Hz (Supplementary Fig. 6b). The 29 Hz closely agrees with direct measurements of 30 Hz, made using another *P. danica* isolate (Christensen-Dalsgaard and Fenchel, 2003), whose cells, compared with our large-size *P. danica* cells, were considerably bigger: 6× larger cell volume with 2.5× longer flagellum. The agreement suggests that the stroke frequency is conserved between different *P. danica* isolates and, perhaps, within the morphotypes of oceanic sHF. The sHF living at perpetually low ≤2 × 10^9^ cells L^−1^ prey concentrations would force them to keep stroke frequencies maximal. The wavelength or stroke motion of our large-size *P. danica* isolate was 2.6 μm (Supplementary Fig. 7a), which equates to a linear speed of 25 cell length s^−1^ (Supplementary Fig. 7c) at 31.5 Hz and 13 cell length s^−1^ at 17 Hz. The latter value is close to 14 cell length s^−1^, which was determined directly in the abundance of prey by microscopic video imaging (Supplementary Video, the 2nd second). Hence, we could conclude that the tractellum model produces realistic propulsion estimates of *P. danica* and, probably, (if all other assumptions hold) of HK too. Because there is no current experimental evidence (to our knowledge) to validate predictions of the pulsellum and trajectellum models (Supplementary Fig. 7c), the predictions of *S. oceanica*, *C. conicella*, aCho and stHK speeds are worthy of future testing.

### Explaining HK dominance in the open ocean

Assuming that the deduced stroke frequencies of the sHF types are maximal and remain constant at lower prey concentrations, we can assess doubling times of the sHF fed on prey in a size range from the bacterioplankton cells with LNA contents (0.055 μm^3^) to the pigmented protists (1.73 μm^3^, comparable in size to sHF) at a range of their oceanic concentrations (Supplementary Table 2 and Supplementary Fig. 2). To make these assessments more realistic, we need to account for energy costs of sampling vast (relative to sHF size) volumes of seawater to intercept sparse prey. Because the larger the cell appendage is, the more energy it requires for operation, we assumed for simplicity that the combined volume of appendages (flagella plus collar) relative to total cell volume (Supplementary Fig. 7b) expressed as a percentage equates to the percentage of cell energy required for their operation. In its turn, the total cell energy equates to the total prey volume required for sHF cell doubling with an assimilation efficiency of 20%, while the other 80% of the captured prey volume is metabolized.

For example, at the model prey concentration of 2.2 × 10^9^ cells L^−1^ (set as the maximal prey abundance), the stHK and *S. oceanica* cells intercept prey every 37 and 10 minutes using 12% and 10% of prey volume (out of 80% metabolized) for propulsion and prey handling, while assimilating 20% of prey volume into predator’s biovolume. At lower prey concentrations, sHF would require more time, i.e. extra energy (reducing predator’s biovolume), to intercept prey. That would slow down the sHF growth and lengthen the doubling time. We set the sHF extinction threshold when their doubling time reaches either 100 days, or fewer days in those cases where a sHF cell spends energy equivalent to 50% of its biovolume on sampling water to intercept its next prey.

**Fig. 8:**
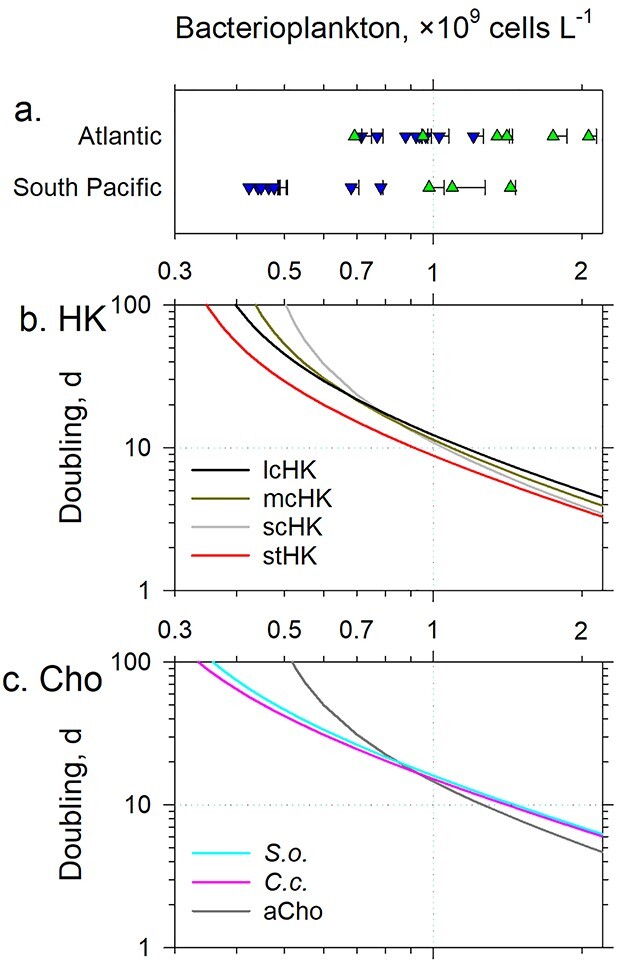
Comparison of the bacterioplankton concentrations measured in the oligotrophic (blue triangles down) and mesotrophic (green triangles up) waters of the Atlantic and South Pacific Oceans (**a**) with bacterioplankton concentrations that could sustain doubling of the seven morphotypes, (**b**) with doubling times of the lcHK, the mcHK, the scHK and the stHK and (**c**) with doubling times of the lCho: *S. oceanica* (*S.o.*), *C. conicella* (*C.c.*) and average aCho as a function of bacterioplankton prey concentration. The bacterioplankton biovolume (0.083 μm^3^), determined in the South Pacific oligotrophic waters, was used in calculations. Error bars in (a) indicate a single standard deviation of the average of concentrations determined at a range of depths in the surface mixed layer. The dotted gridlines aid comparisons.

Using this algorithm, we assessed doubling times of the seven sHF types preying on total bacterioplankton (Fig. 8) at a range of bacterioplankton concentrations from maximal to the ones that led to sHF extinction. While all seven types could double within 30 days at bacterioplankton concentrations found in the South Pacific and Atlantic mesotrophic waters and South Atlantic oligotrophic waters, the scHK and aCho would be unable to live at the bacterioplnkton concentrations found in the South Pacific oligotrophic waters and only *S. oceanica*, *C. conicella* and the stHK could barely survive at the lowest bacterioplankton concentration in the South Pacific oligotrophic waters. The model predictions concur with our observations: the aCho and scHK were practically absent in the South Pacific oligotrophic waters, where bacterioplankton concentrations were <0.5 × 10^9^ cells L^−1^, whereas the *S. oceanica*, *C. conicella*, lcHK and mcHK were present and the stHK numerically dominated the South Pacific oligotrophic waters (Fig. 5, Supplementary Tables 4 and 5). Although the seven and five sHF types could live in the South Atlantic and South Pacific oligotrophic waters, respectively, by preying indiscriminately on bacterioplankton, it is uncertain whether the sHF types could survive by preying selectively on specific groups of bacterioplankton or pigmented protists, as a few observations suggest (Hartmann *et al.*, 2013; Kamennaya *et al.*, 2018).

The model enables us to test feasibility of selective predation by the sHF by comparing the range of observed concentrations of the potential prey groups (Supplementary Table 2) with the range of their respective concentrations which could sustain doubling of the sHF types. It seems that only the highest concentrations of the bacterioplankton with LNA contents and *Prochlorococcus* spp*.*, observed in the South Pacific and Atlantic mesotrophic waters, could sustain doubling of sHF irrespective of their type (Supplementary Fig. 8). Therefore, the dominant bacterioplankton populations are unlikely candidates for selective predation by the sHF. Similarly, only the highest, blooming *Synechococcus* spp*.* concentrations could sustain doubling of the sHF (Supplementary Fig. 9), while the small pigmented protists could only sustain doubling of the sHF when their concentrations are >0.01 × 10^9^ cells L^−1^, atypical even for the South Pacific and Atlantic mesotrophic waters. Although the loricate *C. conicella* and *S. oceanica* seem to capture the small bacterioplankton cells with LNA contents (Supplementary Figs 3 and 8c, e) more efficiently, while the lcHK seem to capture the larger prey like the small pigmented protists (Supplementary Figs 3 and 9c and e) more efficiently, ultimately low ambient prey concentrations drive the sHF into non-selective predation (Fig. 8). Thus, modeling suggests that none of the examined sHF types could survive in the oligotrophic waters by preying selectively, i.e. sHF should be omnivorous.

## CONCLUSIONS

Ubiquity and ocean-scale morphological conservation of the compared sHF morphotypes indicate that their oceanic habitat is a borderless continuum of which the oligotrophic waters are merely the sparser-populated giant parts. Flagella of all examined sHF, except the stHK, suffice to generate a propulsion wave required for sustained locomotion in a line. The predominance of the outlying morphotype, however, suggests that its tiny size and deduced cumbersome tumbling motion is an advantage rather than a handicap, i.e. it is a morphologically “winning design” of the smallest omnivorous planktonic protozoa. At an average concentration of ~500 × 10^3^ cells L^−1^ (Supplementary Table 5c) in >100 m deep surface layer of the open ocean with the area of ~350 × 10^6^ Km^2^, i.e. with >1.9 × 10^25^ cells on Earth, the stHK is a likely candidate to be the most abundant predator on Earth. Although the stHK feeding is clearly successful, as their abundance indicates and modeling results corroborate, precisely how their short tinsel flagellum achieves its effectiveness remains to be determined by experimental studies on living cells.

## Supplementary Material

Supplementary_Information_Revised_fbac031

Supplementary_Video_P-danica_locomotion_fbac031

## References

[ref1] Adl, S. M., Bass, D., Lane, C. E., Lukeš, J., Schoch, C. L., Smirnov, A., Agatha, S., Berney, C. et al. (2019) Revisions to the classification, nomenclature, and diversity of eukaryotes. J. Eukaryotic Microbiol., 66, 4–119. 10.1111/jeu.12691 PMC6492006 30257078

[ref2] Andersen, R. A., Barr, D. J. S., Lynn, D. H., Melkonian, M., Moestrup, Ø. and Sleigh, M. A. (1991) Terminology and nomenclature of the cytoskeletal elements associated with the flagellar/ciliary apparatus in protists. Protoplasma, 164, 1–8.

[ref3] Asadzadeh, S. S., Nielsen, L. T., Andersen, A., Dölger, J., Kiørboe, T., Larsen, P. S. and Walther, J. H. (2019) Hydrodynamic functionality of the lorica in choanoflagellates. J. R. Soc. Interface, 16, 20180478. 30958164 10.1098/rsif.2018.0478 PMC6364640

[ref4] Azam, F., Fenchel, T., Field, J. G., Gray, J. S., Meyer-Reil, L. A. and Thingstad, F. (1983) The ecological role of water-column microbes in the sea. Mar. Ecol. Prog. Ser., 10, 257–263.

[ref5] Caron, D. A. (1983) Technique for enumeration of heterotrophic and phototrophic nanoplankton, using epifluorescence microscopy, and comparison with other procedures. Appl. Environ. Microbiol., 46, 491–498. 16346372 10.1128/aem.46.2.491-498.1983 PMC239428

[ref6] Christensen-Dalsgaard, K. K. and Fenchel, T. (2003) Increased filtration efficiency of attached compared to free-swimming flagellates. Aquatic Microb. Ecol., 33, 77–86.

[ref7] Cynar, F. J., Estep, K. W. and Sieburth, J. M. N. (1985) The detection and characterization of bacteria-sized protists in “protist-free” filtrates and their potential impact on experimental marine ecology. Microb. Ecol., 11, 281–288. 24221498 10.1007/BF02016812

[ref8] De Vargas, C., Audic, S., Henry, N., Decelle, J., Mahé, F., Logares, R., Lara, E., Berney, C. et al. (2015) Eukaryotic plankton diversity in the sunlit ocean. Science, 348, 1261605. 25999516 10.1126/science.1261605

[ref9] Fenchel, T. (1982a) Ecology of heterotrophic microflagellates. I. Some important forms and their functional morpholology. Mar. Ecol. Prog. Ser., 8, 211–223.

[ref10] Fenchel, T. (1982b) Ecology of heterotrophic microflagellates. II. Bioenergetics and growth. Mar. Ecol. Prog. Ser*.*, 8, 225–231.

[ref11] Gomez-Pereira, P. R., Hartmann, M., Grob, C., Tarran, G. A., Martin, A. P., Fuchs, B. M., Scanlan, D. J. and Zubkov, M. V. (2013) Comparable light stimulation of organic nutrient uptake by SAR11 and *Prochlorococcus* in the North Atlantic subtropical gyre. ISME J., 7, 603–614. 23096403 10.1038/ismej.2012.126 PMC3580278

[ref12] Hartmann, M., Zubkov, M. V., Scanlan, D. J. and Lepère, C. (2013) In situ interactions between photosynthetic picoeukaryotes and bacterioplankton in the Atlantic Ocean: evidence for mixotrophy. Environ. Microbiol. Rep., 5, 835–840. 24249292 10.1111/1758-2229.12084

[ref13] Higdon, J. J. (1979) A hydrodynamic analysis of flagellar propulsion. J. Fluid Mech., 90, 685–711.

[ref14] Hoepffner, N. and Haas, L. W. (1990) Electron microscopy of nanoplankton from the North Pacific Central Gyre. J. Phycol., 26, 421–439.

[ref15] Holwill, M. E. J. and Sleigh, M. A. (1967) Propulsion by hispid flagella. J. Exp. Biol., 47, 267–276. 6065814 10.1242/jeb.47.2.267

[ref16] Kamennaya, N. A., Kennaway, G., Fuchs, B. M. and Zubkov, M. V. (2018) Pomacytosis—semi-extracellular phagocytosis of cyanobacteria by the smallest marine algae. PLoS Biol., 16, e2003502. 29304142 10.1371/journal.pbio.2003502 PMC5773223

[ref17] Kendall, A. I. (1923) Bacterial metabolism. Physiol. Rev., 3, 438–455.

[ref18] Leadbeater, B. S. C. (2015) The Choanoflagellates, Evolution, Biology, and Ecology, Cambridge University Press, Cambridge.

[ref19] Marie, D., Partensky, F., Jacquet, S. and Vaulot, D. (1997) Enumeration and cell cycle analysis of natural populations of marine picoplankton by flow cytometry using the nucleic acid stain SYBR Green I. Appl. Environ. Microbiol., 63, 186–193. 16535483 10.1128/aem.63.1.186-193.1997 PMC1389098

[ref20] Mary, I., Heywood, J. L., Fuchs, B. M., Amann, R., Tarran, G. A., Burkill, P. H. and Zubkov, M. V. (2006) SAR11 dominance among metabolically active low nucleic acid bacterioplankton in surface waters along an Atlantic meridional transect. Aquat. Microb. Ecol., 45, 107–113.

[ref21] Massana, R., Terrado, R., Forn, I., Lovejoy, C. and Pedrós-Alió, C. (2006) Distribution and abundance of uncultured heterotrophic flagellates in the world oceans. Environ. Microbiol., 8, 1515–1522. 16913912 10.1111/j.1462-2920.2006.01042.x

[ref23] Nielsen, L. T. and Kiørboe, T. (2021) Foraging trade-offs, flagellar arrangements, and flow architecture of planktonic protists. PNAS, 118, e2009930118. 33431666 10.1073/pnas.2009930118 PMC7826366

[ref24] Nitsche, F., Thomsen, H. A. and Richter, D. J. (2017) Bridging the gap between morphological species and molecular barcodes—exemplified by loricate choanoflagellates. Europ. J. Protistol., 57, 26–37. 10.1016/j.ejop.2016.10.006 28011296

[ref25] Olenina, I., Hajdu, S., Edler, L., Wasmund, N., Busch, S., Göbel, J., Gromisz, S., Huseby, S. et al. (2006) Biovolumes and size-classes of phytoplankton in the Baltic Sea. Baltic Sea Environ. Proc., 106, 144.

[ref26] Pace, N. R. (1997) A molecular view of microbial diversity and the biosphere. Science, 276, 734–740. 9115194 10.1126/science.276.5313.734

[ref27] Patterson, D. J., Nygaard, K., Steinberg, G. and Turley, C. M. (1993) Heterotrophic flagellates and other protists associated with oceanic detritus throughout the water column in the mid North Atlantic. J. Mar. Biol. Assoc. U. K., 73, 67–95.

[ref28] Ramanujan, S. (1914) Modular equations and approximations to π. Q. J. Pure Appl. Math., 45, 350–372.

[ref29] Rice, J., O'Connor, C. D., Sleigh, M. A., Burkill, P. H., Giles, I. G. and Zubkov, M. V. (1997) Fluorescent oligonucleotide rDNA probes that specifically bind to a common nanoflagellate, *Paraphysomonas vestita*. Microbiol., 143, 1717–1727. 10.1099/00221287-143-5-1717 9168621

[ref30] Seeleuthner, Y., Mondy, S., Lombard, V., Carradec, Q., Pelletier, E., Wessner, M., Leconte, J., Mangot, J. F. et al. (2018) Single-cell genomics of multiple uncultured stramenopiles reveals underestimated functional diversity across oceans. Nat. Commun., 9, 310. 29358710 10.1038/s41467-017-02235-3 PMC5778133

[ref31] Sherr, B. F. and Sherr, E. B. (1983) Enumeration of heterotrophic microprotozoa by epifluorescence microscopy. Estuar. Coast. Shelf Sci., 16, 1–7.

[ref32] Sherr, E. B. and Sherr, B. F. (1993) Preservations and storage of samples for enumeration of heterotrophic protists. In Kemp, P. F., Sherr, B. F., Sherr, E. B. and Cole, J. J. (eds.), Handbook of Methods in Aquatic Microbial Ecology, CRC Press, Boca Raton, pp. 207–212.

[ref22] Sieburth, J. McN. (1978) Sea Microbes, Oxford University Press, New York, 491 p.

[ref33] Sieburth, J. M., Smetacek, V. and Lenz, J. (1978) Pelagic ecosystem structure, Heterotrophic compartments of the plankton and their relationship to plankton size fractions. Limnol. Oceanogr., 23, 1256–1263.

[ref34] Sleigh, M. A. (1964) Flagellar movement of the sessile flagellates *Actinomonas*, *Codonosiga*, *Monas* and *Poteriodendron*. Q. J. Microsc. Sci., 105, 405–414.

[ref35] Sleigh, M. A. (1989) Protozoa and Other Protists, Edward Arnold, London.

[ref36] Sleigh, M. A. (1991) Mechanisms of flagellar propulsion. A biologist's view of the relation between structure, motion, and fluid mechanics. Protoplasma, 164, 45–53.

[ref37] Thaler, M. and Lovejoy, C. (2015) Biogeography of heterotrophic flagellate populations indicates the presence of generalist and specialist taxa in the Arctic Ocean. Appl. Environ. Microbiol., 81, 2137–2148. 25595764 10.1128/AEM.02737-14 PMC4345384

[ref38] Thomsen, H. A., Hara, S. and Østergaard, J. B. (2021) Loricate choanoflagellates (Acanthoecida) from warm water seas. IX. *Coronoeca* gen. nov., *Polyfibula* Manton and spiny forms of *Parvicorbicula* Deflandre. Europ. J. Protistol., 81, 125826. 10.1016/j.ejop.2021.125826 34399128

[ref39] Thomsen, H. A., Kamennaya, N. A., Zubkov, M. V. and Østergaard, J. B. (2020) Loricate choanoflagellates (Acanthoecida) from warm water seas. VII. *Calotheca* Thomsen and Moestrup, *Stephanacantha* Thomsen and *Syndetophyllum* Thomsen and Moestrup. Europ. J. Protistol., 76, 125728. 10.1016/j.ejop.2020.125728 32682306

[ref40] Vørs, N., Buck, K. R., Chavez, F. P., Eikrem, W., Hansen, L. E., Østergaard, J. B. and Thomsen, H. A. (1995) Nanoplankton of the equatorial Pacific with emphasis on the heterotrophic protists. Deep-Sea Res. II Top. Stud. Oceanogr., 42, 585–602.

[ref41] Zubkov, M. V., Burkill, P. H. and Topping, J. N. (2007) Flow cytometric enumeration of DNA-stained oceanic planktonic protists. J. Plankton Res., 29, 79–86.

[ref42] Zubkov, M. V., Sazhin, A. F. and Flint, M. V. (1992) The microplankton organisms at the oxic-anoxic interface in the pelagial of the Black Sea. FEMS Microbiol. Ecol., 10, 245–250.

[ref43] Zubkov, M. V. and Sleigh, M. A. (2005) Assimilation efficiency of *Vibrio* bacterial protein biomass by the flagellate *Pteridomonas*, assessment using flow cytometric sorting. FEMS Microbiol. Ecol., 54, 281–286. 16332326 10.1016/j.femsec.2005.04.001

[ref44] Zubkov, M. V., Sleigh, M. A., Burkill, P. H. and Leakey, R. J. (2000) Picoplankton community structure on the Atlantic Meridional Transect, a comparison between seasons. Prog. Oceanogr., 45, 369–386.

[ref45] Zubkov, M. V., Sleigh, M. A., Tarran, G. A., Burkill, P. H. and Leakey, R. J. (1998) Picoplanktonic community structure on an Atlantic transect from 50°N to 50°S. Deep-Sea Res. I Oceanogr. Res. Pap., 45, 1339–1355.

[ref46] Zubkov, M. V. and Tarran, G. A. (2008) High bacterivory by the smallest phytoplankton in the North Atlantic Ocean. Nature, 455, 224–226. 18690208 10.1038/nature07236

