# Supplementary material for: Notable predominant morphology of the smallest most abundant protozoa of the open ocean revealed by electron microscopy
Source: J Plankton Res. 2022 Jul 7;44(4):542–58. doi: 10.1093/plankt/fbac031 (PMC9310263; doi:10.1093/plankt/fbac031)
Supplement: Supplementary_Information_Revised_fbac031 [file supplementary_information_revised_fbac031.docx]

**Supplementary information** for the manuscript intitled

**Notable predominant morphology of the smallest most abundant protozoa of the open ocean revealed by electron microscopy**

by Nina A. Kamennaya, Gabrielle Kennaway, Michael A. Sleigh, Mikhail V. Zubkov

It comprises **9** Supplementary Figures, **12** Supplementary Tables with Supplementary Statistical Analyses and **1** Supplementary Video.

**Supplementary Figure 1.** Contour plots of temperature (a, ^o^C), salinity (b, kg m^-3^) and estimated chlorophyll *a* concentration (c, µg *chl a* kg^-1^ seawater) in the upper 300 m layer along the longitudinal transect across the South Pacific Ocean from temperate waters east of the North Island of New Zealand to near Easter Island. In c grey lines indicate the vertical profiles and cyan diamonds indicate the locations and depths of the samples taken for sHF morphotype analysis.

**Supplementary Figure 2.** Contour plots of concentrations of total bacterioplankton (a, ×10^9^ cells L^-1^), the smallest pigmented protist (b, ×10^6^ cells L^-1^) and the small heterotrophic flagellates (sHF, c, ×10^6^ cells L^-1^) in the upper 300 m layer along the longitudinal transect in the South Pacific Ocean. Black circles and cyan diamonds indicate depths of sample collection for microbial enumeration and analysis of sHF morphology, respectively.

**Supplementary Figure 3.** Characteristic flow cytometric signatures (left column) of the concentrated, DNA-stained oceanic microbes used for targeted flow sorting of the low nucleic acid containing bacteria (LNA) predominantly comprised of thin, bended long rods (characteristic cell morphology of the SAR11 group), *Prochlorococcus* spp. (*Pro*) and *Synechococcus* spp. (*Syn*) cyanobacteria and the small, pigmented protists (Alg) with their corresponding SEM micrographs (right column) to demonstrate sorting purity.

a. A log plot of 10^3^ dots of Hoechst-DNA fluorescence above the set threshold (FL1) excited by the first 355 nm laser versus shallow angle or forward light scatter (FSC) showing the bacterioplankton population with a subpopulation of LNA cells with lower DNA fluorescence, i.e. content, indicated by the blue oval.

b. A log plot of 10^3^ dots of Hoechst-DNA fluorescence (FL1) versus red fluorescence (FL7) excited by the second 488nm laser showing the bacterioplankton with a subpopulation of *Pro* cells with higher chlorophyll autofluorescence, indicated by the blue oval.

c. A log plot of 10^5^ dots of Hoechst-DNA fluorescence (FL1) versus orange fluorescence (FL6) excited by the second 488nm laser showing the bacterioplankton with a minor subpopulation of *Syn* cells (blue oval) with higher phycoerythrin autofluorescence.

d. A log plot of 2×10^6^ dots of Hoechst-DNA fluorescence (FL1) versus red fluorescence (FL4) excited by the first laser showing the bacterioplankton and smallest protists with the dominant population of algal cells with higher chlorophyll autofluorescence, indicated by the blue oval. Notice the scale change to amplify the targeted population and 1.0-µm reference beads (cyan oval).

|  | LNA |
| --- | --- |
|  |  |
|  | *Pro* |
|  |  |
|  | *Syn* |
|  |  |
|  | Alg |
|  |  |

**Supplementary Figure 4.** Examples of the flow sorted *Pteridomonas danica* (*P. danica*) isolates: large (*P. danica* L), medium (*P. danica* M) and small (*P. danica* S). *Pd*-L (a. & b.) demonstrate the cluster size of the sorted cells and sorting purity of the targeted populations, respectively.

| *P. danica* L, a. | *P. danica* L, b. |
| --- | --- |
| 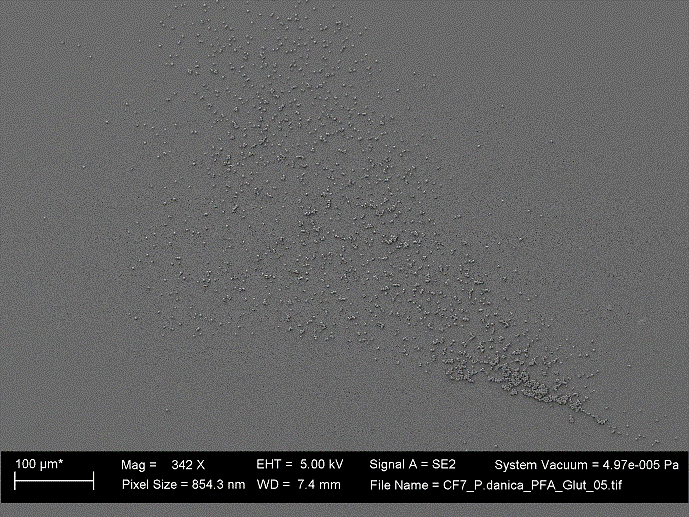 |  |
| *P. danica* L, c. | *P. danica* L, d. |
|  |  |
| *P. danica* M, a. | *P. danica* M, b. |
|  |  |
| *P. danica* S, a. | *P. danica* S, b. |
|  |  |

**Supplementary Figure 5.** Additional examples of the flow sorted morphotypes of heterokonts: the long caudal heterokont (lcHK), the medium caudal heterokont (mcHK), the short caudal heterokont (scHK) and the short tinsel heterokont (stHK), respectively. Details of intracellular and mastigoneme organization of the stHK are also presented (stHK, c-d).

| lcHK, a. | lcHK, b. |
| --- | --- |
|  |  |
| mcHK. | scHK. |
|  |  |
| stHK, a. | stHK, b. |
|  |  |
| stHK, c. | stHK, d. |
|  | 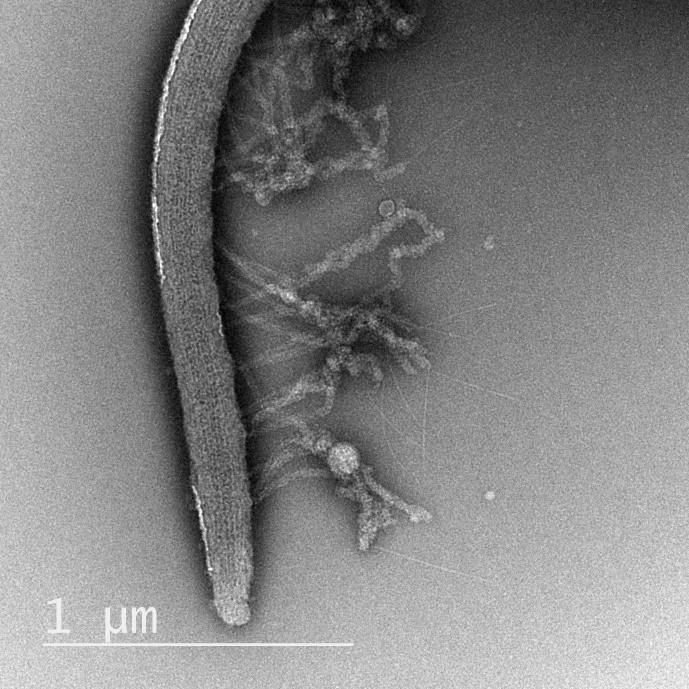 |

**Supplementary Figure 6.** Comparison of (a) mean cell size (length and width), and (b) dimensions and stroke frequencies of the propulsive flagellum of the *Pteridomonas danica* isolate with large cells (P.d.) versus the representative loricate choanoflagellate species, *Stephanacantha oceanica* (S.o.), *Coronoeca conicella* (C.c.) and average aloricate choanoflagellates (aCho), as well as the long caudal heterokont (lcHK), the medium caudal heterokont (mcHK), the short caudal heterokont (scHK) and the short tinsel heterokont (stHK) morphotypes in the open ocean. Cell dimensions are corrected for drying shrinkage apart from the flagellar length.


**Supplementary** **Figure 7.** Comparison of the cell interception cross-section and stroke motion (a), biovolume and doubling time (b), absolute and relative population speeds (c) of the large cell *Pteridomonas danica* isolate (*P.d.*) versus the representative loricate choanoflagellate species, *Stephanacantha oceanica* (S.o.), *Coronoeca conicella* (C.c.) and average aloricate choanoflagellates (aCho), as well as the long caudal heterokont (lcHK), the medium caudal heterokont (mcHK), the short caudal heterokont (scHK) and the short tinsel heterokont (stHK) morphotypes in the open ocean. The black and grey parts of the bar indicate the flagellar/collar and cell parts of the cross-section (a) and biovolume (b).

**Supplementary** **Figure 8.** Comparison of the concentrations of low nucleic acid containing bacteria (LNA) and *Prochlorococcus* spp. (*Pro*) measured in the oligotrophic (blue triangles down) and mesotrophic (green triangles up) waters of the Atlantic and South Pacific Oceans (a, b) with their concentrations that could sustain doubling of the seven sHF morphotypes (left and right column), (c, d) with doubling times of the long caudal heterokont (lcHK), the medium caudal heterokont (mcHK), the short caudal heterokont (scHK) and the short tinsel heterokont (stHK) and (e, f) with doubling times of the choanoflagellates (Cho): *Stephanacantha oceanica* (*S.o.*), *Coronoeca conicella* (*C.c.*) and average aloricate choanoflagellates (aCho) as a function of prey concentration. The LNA (0.055 µm^3^) and *Pro* (0.132 µm^3^) biovolumes, determined in the South Pacific oligotrophic waters, were used in calculations. Error bars (a, b) indicate a single standard deviation of the average of concentrations determined at a range of depths in the surface mixed layer. The dotted gridlines aid comparisons.

**Supplementary** **Figure 9.** Comparison of the concentrations of *Synechococcus* spp. (*Syn*) and the small pigmented protists (Alg) measured in the oligotrophic (blue triangles down) and mesotrophic (green triangles up) waters of the Atlantic and South Pacific Oceans (a, b) with their concentrations that could sustain doubling of the seven sHF morphotypes (left and right column), (c, d) with doubling times of the long caudal heterokont (lcHK), the medium caudal heterokont (mcHK), the short caudal heterokont (scHK) and the short tinsel heterokont (stHK) and (e, f) with doubling times of the choanoflagellates (Cho): *Stephanacantha oceanica* (*S.o.*), *Coronoeca conicella* (*C.c.*) and average aloricate choanoflagellates (aCho) as a function of prey concentration. The *Syn* (0.77 µm^3^) and small pigmented protist (2.72 µm^3^) biovolumes, determined in the South Pacific mesotrophic waters, were used in calculations. Error bars (a, b) indicate a single standard deviation of the average of concentrations determined at a range of depths in the surface mixed layer. The terminal small circles in (d) indicate the extinction threshold points, when 50% of predator biovolume was spent on sampling water to intercept next prey. The dotted gridlines aid comparisons.

**Supplementary Table 1.** Date, station identifier in the South Pacific and Atlantic mesotrophic and oligotrophic waters (SPacM, AtlM, SPacO and AtlO, respectively), geographic location (latitude and longitude), depth (m) and temperature (°C) of samples from which the small heterotrophic flagellates (sHF) were flow sorted and imaged.

| Date | Waters_  Station | Lat.,  ′* | Long.,  ′W | Depth, m | Temp, °C |
| --- | --- | --- | --- | --- | --- |
| 23.01.16 | SPacM_1 | -39.000 | 170.000 | 40 | 15.1 |
| 21.01.16 | _2 | -39.000 | 160.001 | 20 | 14.4 |
| 18.01.16 | _3 | -38.998 | 150.000 | 50 | 16.7 |
| 15.01.16 | SPacO_1 | -39.310 | 139.810 | 40 | 17.9 |
| 13.01.16 | _2 | -36.362 | 132.675 | 75 | 16.3 |
| 11.01.16 | _3 | -33.504 | 126.008 | 125 | 16.1 |
| 11.01.16 | _4 | -33.500 | 125.999 | 125 | 16.2 |
| 04.01.16 | _5 | -23.500 | 110.049 | 175 | 20.1 |
| 03.12.16 | AtlM_1 | +23.608 | 20.714 | 25 | 23.1 |
| 09.10.17 | _2 | +7.749 | 26.896 | 20 | 28.8 |
| 11.10.17 | _3 | +2.439 | 25.612 | 20 | 27.7 |
| 17.10.17 | AtlO_1 | -15.961 | 25.002 | 20 | 24.1 |
| 19.10.17 | _2 | -20.746 | 25.064 | 20 | 22.4 |
| 20.10.14 | _3 | -20.856 | 25.052 | 155 | 21.4 |
| 21.10.17 | _4 | -27.550 | 25.194 | 20 | 19.9 |
| 22.10.14 | _5 | -27.747 | 25.031 | 125 | 18.9 |
| 23.10.17 | _6 | -33.129 | 26.840 | 20 | 17.6 |
| 24.10.14 | _7 | -34.69 | 27.429 | 20 | 16.7 |
| 25.10.14 | AtlM_4 | -37.893 | 28.768 | 20 | 14.0 |
| 26.10.17 | _5 | -42.128 | 30.417 | 20 | 10.8 |
| 28.10.14 | _6 | -43.003 | 37.141 | 20 | 11.6 |

*, Latitudes: North (+), South (-)

**Supplementary Table 2.** Station identifier (St-Id) in in the South Pacific and Atlantic mesotrophic and oligotrophic waters (SPacM, AtlM, SPacO and AtlO, respectively) and corresponding concentrations of *Synechococcus* spp. (*Syn*, ×10^9^ cells L^-1^) and *Prochlorococcus* spp. (*Pro*, ×10^9^ cells L^-1^) cyanobacteria, low nucleic acid containing bacteria (LNA, ×10^9^ cells L^-1^), total bacterioplankton (Bpl, ×10^9^ cells L^-1^) as well as the concentrations of the total small pigmented protists (Alg, ×10^6^ cells L^-1^) and small heterotrophic flagellates (sHF, ×10^6^ cells L^-1^). The average (Avg) concentrations for the surface mixed layer, corresponding standard deviations (SD) and the number of bacterioplankton and protist analyses (n).

|  | *Syn* |  | *Pro* |  | LNA | | Bpl |  |  | Alg |  | sHF |  |  |
| --- | --- | --- | --- | --- | --- | --- | --- | --- | --- | --- | --- | --- | --- | --- |
| St-Id | Avg | SD | Avg | SD | Avg | SD | Avg | SD | n | Avg | SD | Avg | SD | n |
| SPacM_1 | 0.023 | 0.010 | ND |  | 0.429 | 0.038 | 0.981 | 0.072 | 4 | 3.666 | 0.084 | 0.349 | 0.128 | 4 |
| _2 | 0.010 | 0.001 | 0.301 | 0.010 | 0.668 | 0.015 | 1.434 | 0.034 | 4 | 4.559 | 0.495 | 1.317 | 0.188 | 4 |
| _3 | 0.007 | 0.006 | 0.177 | 0.129 | 0.564 | 0.046 | 1.093 | 0.181 | 3 | 4.307 | 1.923 | 0.416 | 0.263 | 3 |
| SPacO_1 | ND |  | 0.164 | 0.002 | 0.496 | 0.007 | 0.783 | 0.007 | 3 | 3.030 | 1.810 | 0.537 | 0.171 | 2 |
| _2 | 0.006 | 0.001 | 0.143 | 0.002 | 0.353 | 0.014 | 0.682 | 0.025 | 2 | 1.870 | 0.090 | 0.651 | 0.016 | 2 |
| _3 | 0.0003 | 0.0001 | 0.086 | 0.002 | 0.234 | 0.015 | 0.477 | 0.013 | 4 | 1.206 | 0.127 | 0.392 | 0.065 | 4 |
| _4 | ND |  | 0.053 | 0.009 | 0.227 | 0.021 | 0.449 | 0.056 | 3 | 0.765 | 0.076 | 0.314 | 0.078 | 2 |
| _5 | ND |  | 0.067 | 0.011 | 0.254 | 0.014 | 0.477 | 0.031 | 7 | 0.889 | 0.095 | 0.186 | 0.038 | 7 |
| AtlM_1 | 0.029 | 0.005 | 0.620 | 0.033 | 0.853 | 0.074 | 1.750 | 0.117 | 4 | 1.514 | 0.114 | 1.332 | 0.089 | 6 |
| _2 | 0.027 | 0.001 | 0.383 | 0.30 | 0.650 | 0.042 | 1.347 | 0.081 | 2 | 1.139 | 0.040 | 1.061 | 0.066 | 2 |
| _3 | 0.034 | 0.001 | 0.541 | 0.022 | 0.658 | 0.050 | 1.410 | 0.035 | 2 | 4.465 | 0.152 | 1.888 | 0.008 | 3 |
| AtlO_1 | 0.0047 | 0.001 | 0.409 | 0.030 | 0.607 | 0.050 | 1.207 | 0.055 | 4 | 2.040 | 0.211 | 0.900 | 0.187 | 5 |
| _2 | 0.0040 | 0.0004 | 0.290 | 0.004 | 0.523 | 0.018 | 0.966 | 0.025 | 8 | 1.641 | 0.360 | 0.952 | 0.166 | 8 |
| _3 | 0.0037 | 0.0007 | 0.323 | 0.007 | 0.527 | 0.040 | 1.027 | 0.047 | 3 | 0.626 | 0.159 | 0.845 | 0.138 | 3 |
| _4 | 0.0023 | 0.0003 | 0.277 | 0.015 | 0.501 | 0.006 | 0.938 | 0.013 | 6 | 0.598 | 0.142 | 0.793 | 0.196 | 9 |
| _5 | 0.0024 | 0.0003 | 0.251 | 0.015 | 0.520 | 0.022 | 0.923 | 0.024 | 7 | 1.004 | 0.249 | 0.793 | 0.196 | 9 |
| _6 | 0.0021 | 0.0004 | 0172 | 0.006 | 0.460 | 0.019 | 0.770 | 0.020 | 8 | 0.830 | 0.106 | 0.763 | 0.198 | 9 |
| _7 | 0.0025 | 0.0006 | 0.160 | 0.011 | 0.394 | 0.019 | 0.715 | 0.034 | 3 | 0.887 | 0.085 | 0.844 | 0.369 | 4 |
| AtlM_4 | 0.007 | 0.0007 | 0.247 | 0.010 | 0.483 | 0.014 | 0.953 | 0.022 | 6 | 2.361 | 0.149 | 0.919 | 0.116 | 7 |
| _5 | 0.578 | 0.017 | ND |  | 1.116 | 0.032 | 2.065 | 0.076 | 3 | 15.15 | 0.31 | 6.640 | 1.280 | 3 |
| _6 | 0.0076 | 0.0008 | ND |  | 0.232 | 0.015 | 0.693 | 0.024 | 7 | 14.89 | 2.15 | 1.310 | 0.124 | 5 |

ND – not determined, i.e. abundance below the detection limit of the flow cytometry method used.

**Supplementary Table 3.** Number of sHF cells sorted at each station in the South Pacific mesotrophic and oligotrophic waters (SPacM and SPacO, respectively), the number and percentage of photographed cells counted using lower resolution SEM.

| Station | # cells sorted | # cell counted | % cell counted |
| --- | --- | --- | --- |
| SPacM_1 | 965 | 448 | 46 |
| _2 | 2121 | 149 | 7 |
| _3 | 660 | 71 | 11 |
| SPacO_1 | 1250 | 425 | 34 |
| _2 | 4000 | 824 | 21 |
| _3 | 3286 | 444 | 14 |
| _4 | 1260 | 297 | 24 |
| _5 | 540 | 44 | 8 |

**Supplementary Table 4.** Absolute (a) and relative (b) number of the analysed small heterotrophic flagellates (sHF), largely comprised of the two main morphotypes: heterokonts (HK) and choanoflagellates (Cho), flow sorted from samples collected in the South Pacific and Atlantic mesotrophic and oligotrophic waters (SPacM, AtlM, SPacO and AtlO, respectively) and examined at higher resolution. The heterokonts were split into the short tinsel heterokont (stHK), short caudal heterokont (scHK), medium caudal heterokont (mcHK) and long caudal heterokont (lcHK) morphotypes and the choanoflagellates were split into the loricate (lCho) and aloricate (aCho) morphotypes.

a. Number of analysed cells

| Waters | sHF | **HK** | stHK | scHK | mcHK | lcHK | **Cho** | lCho | aCho |
| --- | --- | --- | --- | --- | --- | --- | --- | --- | --- |
| SPacO | 224 | **120** | 97 | 4 | 9 | 10 | **104** | 82 | 22 |
| SPacM | 273 | **232** | 94 | 58 | 56 | 24 | **41** | 24 | 17 |
| AtlO | 70 | **59** | 32 | 10 | 8 | 9 | **11** | 7 | 4 |
| AtlM | 112 | **91** | 62 | 8 | 12 | 9 | **21** | 18 | 3 |

b. Percentage of total sHF, %

| Waters | **HK** | stHK | scHK | mcHK | lcHK | **Cho** | lCho | aCho |
| --- | --- | --- | --- | --- | --- | --- | --- | --- |
| SPacO | **53.6** | 43.3 | 1.8 | 4.0 | 4.5 | **46.4** | 36.6 | 9.8 |
| SPacM | **85.0** | 34.4 | 21.2 | 20.5 | 8.8 | **15.0** | 16.1 | 2.7 |
| AtlO | **84.3** | 45.7 | 14.3 | 11.4 | 12.9 | **15.7** | 10.0 | 5.7 |
| AtlM | **81.3** | 55.4 | 7.2 | 10.7 | 8.0 | **18.7** | 16.1 | 2.7 |

**Supplementary Table 5.** Absolute (a) and relative (b) numbers of the counted small heterotrophic flagellates (sHF) of the two main types: heterokonts (HK) and choanoflagellates (Cho). The absolute abundance (c), biomass (d) and relative biomass (e) of the smallest heterotrophic flagellates, flow sorted from samples collected in the South Pacific mesotrophic (SPacM) and oligotrophic (SPacO) waters and examined at higher resolution The heterokonts were split into the short tinsel heterokont (stHK), short caudal heterokont (scHK), medium caudal heterokont (mcHK) and long caudal heterokont (lcHK) morphotypes and the choanoflagellates were split into the loricate (lCho) and aloricate (aCho) morphotypes.

a. Number of counted cells

| Waters | St. | sHF | **HK** | stHK | scHK | mcHK | lcHK | **Cho** | lCho | aCho |
| --- | --- | --- | --- | --- | --- | --- | --- | --- | --- | --- |
| SPacM | 1 | 406 | **368** | 296 | 20 | 49 | 3 | **34** | 22 | 12 |
|  | 2 | 145 | **141** | 105 | 21 | 15 | 0 | **1** | 1 | 0 |
|  | 3 | 61 | **52** | 36 | 5 | 7 | 4 | **4** | 4 | 0 |
| SPacO | 1 | 293 | **202** | 128 | 16 | 19 | 39 | **83** | 45 | 38 |
|  | 2 | 710 | **610** | 554 | 6 | 20 | 30 | **93** | 93 | 0 |
|  | 3 | 397 | **355** | 278 | 9 | 39 | 29 | **23** | 23 | 0 |
|  | 4 | 272 | **249** | 216 | 9 | 16 | 8 | **8** | 8 | 0 |

b. Relative numbers of counted cells, %

| Waters | St. | **HK** | stHK | scHK | mcHK | lcHK | **Cho** | lCho | aCho |
| --- | --- | --- | --- | --- | --- | --- | --- | --- | --- |
| SPacM | 1 | **90.6** | 72.9 | 4.9 | 12.1 | 0.7 | **8.4** | 5.4 | 3.0 |
|  | 2 | **97.2** | 72.4 | 14.5 | 10.4 | 0 | **0.7** | 0.7 | 0 |
|  | 3 | **85.3** | 59.0 | 8.2 | 11.5 | 6.6 | **6.6** | 6.6 | 0 |
| SPacO | 1 | **68.9** | 43.7 | 5.5 | 6.5 | 13.3 | **28.3** | 15.4 | 13.0 |
|  | 2 | **85.9** | 78.0 | 0.9 | 2.8 | 4.2 | **13.1** | 13.1 | 0 |
|  | 3 | **89.4** | 70.0 | 2.3 | 9.8 | 7.3 | **5.8** | 5.8 | 0 |
|  | 4 | **91.5** | 79.4 | 3.3 | 5.9 | 2.9 | **2.9** | 2.9 | 0 |

c. Absolute numbers, ×10^3^ cells L^-1^

| Waters | St. | **HK** | stHK | scHK | mcHK | lcHK | **Cho** | lCho | aCho |
| --- | --- | --- | --- | --- | --- | --- | --- | --- | --- |
| SPacM | 1 | **474** | 382 | 25 | 64 | 4 | **44** | 28 | 16 |
|  | 2 | **1467** | 1092 | 218 | 156 | 0 | **10** | 10 | 0 |
|  | 3 | **608** | 421 | 58 | 82 | 47 | **47** | 47 | 0 |
| SPacO | 1 | **569** | 361 | 45 | 54 | 110 | **234** | 127 | 107 |
|  | 2 | **806** | 732 | 8 | 26 | 40 | **123** | 123 | 0 |
|  | 3 | **449** | 352 | 11 | 49 | 37 | **29** | 29 | 0 |
|  | 4 | **464** | 403 | 17 | 30 | 15 | **15** | 15 | 0 |

d. Absolute biomass, ng C L^-1^

| Waters | St. | **HK** | stHK | scHK | mcHK | lcHK | **Cho** | lCho | aCho |
| --- | --- | --- | --- | --- | --- | --- | --- | --- | --- |
| SPacM | 1 | **225** | 162 | 12 | 46 | 4 | **107** | 88 | 20 |
|  | 2 | **684** | 464 | 107 | 114 | 0 | **32** | 32 | 0 |
|  | 3 | **317** | 179 | 29 | 60 | 50 | **144** | 144 | 0 |
| SPacO | 1 | **332** | 153 | 22 | 39 | 118 | **527** | 392 | 135 |
|  | 2 | **376** | 311 | 4 | 19 | 42 | **380** | 380 | 0 |
|  | 3 | **230** | 149 | 6 | 36 | 39 | **90** | 90 | 0 |
|  | 4 | **217** | 171 | 8 | 22 | 16 | **46** | 46 | 0 |

e. Relative biomass, %

| Waters | St. | **HK** | stHK | scHK | mcHK | lcHK | **Cho** | lCho | aCho |
| --- | --- | --- | --- | --- | --- | --- | --- | --- | --- |
| SPacM | 1 | **67.1** | 48.3 | 3.7 | 13.8 | 1.2 | **32.0** | 26.1 | 5.8 |
|  | 2 | **93.5** | 63.4 | 14.6 | 15.6 | 0 | **4.4** | 4.4 | 0 |
|  | 3 | **63.1** | 35.6 | 5.7 | 11.9 | 10.0 | **28.7** | 28.7 | 0 |
| SPacO | 1 | **37.6** | 17.4 | 2.5 | 4.4 | 13.3 | **59.7** | 44.4 | 15.3 |
|  | 2 | **49.3** | 40.7 | 0.5 | 2.5 | 5.6 | **49.7** | 49.7 | 0 |
|  | 3 | **68.5** | 44.4 | 1.7 | 10.7 | 11.7 | **26.8** | 26.8 | 0 |
|  | 4 | **77.9** | 61.4 | 3.0 | 7.8 | 5.7 | **16.6** | 16.6 | 0 |

**Supplementary Table 6.** Mean cell (length and width) and flagella (tinsel, non-tinsel or caudal and its whip) dimensions and their standard deviations (SD) of the choanoflagellate (Cho) representative species, *Coronoeca conicella* (*C.c.*), *Stephanacantha oceanica* (*S.o.*), average loricate and aloricate choanoflagellates (lCho and aCho, respectively) as well as the long caudal heterokont (lcHK), the medium caudal heterokont (mcHK), the short caudal heterokont (scHK) and the short tinsel heterokont (stHK) in the open ocean plus of the short tinsel heterokont morphotype at the station SPacO_3 (stHK^#^) in the South Pacific (sub)tropical gyre and the three isolates of *Pteridomonas danica* with small, medium and large cells (*P.d.* S, *P.d.* M, *P.d.* L, respectively). Cell dimensions are direct measurements, uncorrected for drying shrinkage. Statistical tests are the equal variance F-test and the equal mean t-test. The critical P-value of <0.05 was used to signify difference between the vertically adjacent means and variances presented as SD, i. e. the symbol < if the top value is significantly lower than the bottom value, the symbol > if the top value is significantly higher than the bottom value and the symbol = if the difference between the two values is insignificant. Relative difference between means is shown as a percentage (%) of the difference between means divided by the lower of the two means.

| Morpho-type | | Length, μm | | Width, μm | |  | Tinsel, μm | |  | Non-tinsel, μm | | | Whip, μm | |  |
| --- | --- | --- | --- | --- | --- | --- | --- | --- | --- | --- | --- | --- | --- | --- | --- |
|  |  | Mean | SD | Mean | SD | n | Mean | SD | n | Mean | SD | n | Mean | SD | n |
| Cho | *C.c.* | 3.74 | 0.64 | 1.79 | 0.23 | 14 |  |  |  | 9.76 | 1.87 | 9 | 2.46 | 0.94 | 5 |
|  |  | = 0.5% | = | = 6% | = |  |  |  |  | = 14% | = |  | = 64% | = |  |
|  | *S.o.* | 3.72 | 0.78 | 1.89 | 0.36 | 21 |  |  |  | 11.1 | 1.97 | 16 | 1.5 | 0.50 | 3 |
|  |  | = 3% | < | = 8% |  |  |  |  |  | = 4% | < |  | = 39% | < |  |
|  | lCho | 3.62 | 1.09 | 2.03 | 0.45 | 131 |  |  |  | 11.6 | 4.03 | 64 | 2.08 | 1.23 | 23 |
|  |  | > 64% | > | > 38% | > |  |  |  |  | > 61% | > |  | = 26% | = |  |
|  | aCho | 2.21 | 0.41 | 1.47 | 0.23 | 46 |  |  |  | 7.23 | 1.13 | 37 | 1.65 | 0.89 | 22 |
|  |  | = 15% | = | = 4% | < |  |  |  |  |  |  |  |  |  |  |
| HK | lcHK | 1.93 | 0.34 | 1.52 | 0.30 | 52 | 9.86 | 1.72 | 35 | 3.28 | 0.68 | 47 | 1.46 | 0.66 | 25 |
|  |  | > 7% | = | > 14% | = |  | > 11% | = |  | > 84% | > |  | = 6% | = |  |
|  | mcHK | 1.79 | 0.37 | 1.34 | 0.25 | 85 | 8.89 | 1.72 | 59 | 1.78 | 0.47 | 50 | 1.37 | 0.48 | 36 |
|  |  | > 26% | > | > 21% | > |  | > 8% | > |  | >153% | > |  | = 2% | = |  |
|  | scHK | 1.42 | 0.23 | 1.11 | 0.17 | 80 | 8.20 | 1.08 | 76 | 0.71 | 0.22 | 37 | 1.34 | 0.52 | 36 |
|  |  | > 5% | = | = 3% | < |  | >138% | > |  | < 71% | > |  | >101% | > |  |
|  | stHK | 1.36 | 0.25 | 1.14 | 0.21 | 285 | 3.45 | 0.43 | 241 | 1.21 | 0.27 | 210 | 0.67 | 0.14 | 153 |
|  | stHK^#^ | 1.30 | 0.20 | 1.10 | 0.17 | 78 | 3.66 | 0.35 | 77 | 1.36 | 0.22 | 67 | 0.71 | 0.16 | 50 |
|  |  | < 22% | = | < 33% | = |  | < 78% | < |  |  |  |  |  |  |  |
| *P.d.* | S | 1.58 | 0.15 | 1.45 | 0.14 | 28 | 6.51 | 0.63 | 27 |  |  |  |  |  |  |
|  |  | < 5% | = | < 6% | = |  | < 6% | = |  |  |  |  |  |  |  |
|  | M | 1.66 | 0.16 | 1.54 | 0.14 | 58 | 6.89 | 0.68 | 56 |  |  |  |  |  |  |
|  |  | < 56% | = | < 54% | < |  | < 22% | < |  |  |  |  |  |  |  |
|  | L | 2.58 | 0.21 | 2.38 | 0.21 | 24 | 8.40 | 1.16 | 22 |  |  |  |  |  |  |

n – number of measurements.

**Supplementary statistical analyses for a****ssessing similarity of the heterokont morphotypes at the ocean-scale**

To examine that the assessments were not biased by SEM- and TEM-based measurements we compared dimensions of the short tinsel heterokont (stHK) (**Supplementary Table 7**) at the SPacO_3 station in the South Pacific oligotrophic waters (**Supplementary Table 1**), at which we had measured a sufficient number of specimens. Because the comparison showed statistical similarity of all measured dimensions, we pooled the SEM- and TEM-based measurements together.

**Supplementary Table 7.** Comparison of cell (length and width) and flagella (tinsel, non-tinsel or caudal and whip) sizes of the short tinsel heterokont (stHK) morphotype sampled at the station SPacO_3 and analysed using scanning and transmission electron microscopy (SEM and TEM, respectively). Cell dimensions are direct measurements, uncorrected for drying shrinkage. Statistical tests are the equal variance F-test and the equal mean t-test (t_t_). The critical P-value of <0.05 was used to signify difference, i.e. the symbol < if the left mean is significantly lower than the right mean, the symbol > if the left mean is significantly higher than the right mean and the symbol = if the difference between the two means is insignificant. Relative difference between means is shown as a percentage (%) of the difference between the means divided by the mean of the lower value, a negative sign indicates that the left mean is lower than the right mean.

|  | SEM |  |  |  |  | TEM |  |  |
| --- | --- | --- | --- | --- | --- | --- | --- | --- |
| Size, μm | Mean | SD | n | t_t_ | % | Mean | SD | n |
| Length | 1.30 | 0.17 | 62 | = | 0.1 | 1.30 | 0.28 | 16 |
| Width | 1.10 | 0.15 | 62 | = | 1.7 | 1.08 | 0.23 | 16 |
| Tinsel | 3.63 | 0.30 | 62 | = | -4.1 | 3.78 | 0.50 | 15 |
| Caudal | 1.36 | 0.21 | 56 | = | -0.4 | 1.37 | 0.29 | 11 |
| Whip | 0.70 | 0.16 | 39 | = | -3.2 | 0.73 | 0.15 | 11 |

Sampling the Atlantic oligotrophic (AtlO) and mesotrophic (AtlM) waters in 2014 and 2017 gave us an opportunity to assess reproducibility of morphometric measurements of the short tinsel heterotroph (stHK) (**Supplementary Table 8**), owing to their abundance (**Fig. 5; Supplementary Tables 4-5**). In the AtlO of the five dimensions measured only the length of the caudal flagellum was statistically different between the two years. The difference could be due to a limited number of measurements (n=3) in 2017 and hence should be treated with caution. In the AtlM two dimensions: the cell width and the length of the tinsel flagellum, showed significant difference but of only 14.3% and 9%, respectively. Because heterokont cells divide longitudinally, the difference in width should reflect the difference in growth of the compared stHK populations. Compared with similarity of the other three dimensions the difference in the tinsel length seems insufficient to consider that the stHK populations sampled in 2014 and 2017 were different. Consequently, we combined the data, collected in the same region on different cruises (**Supplementary Table 1**).

**Supplementary Table 8.** Comparison of cell (length and width) and flagella (tinsel, caudal and whip) sizes of the short tinsel heterokont morphotype (stHK) in the Atlantic oligotrophic (AtlO) and mesotrophic (AtlM) waters in 2014 and 2017. Cell dimensions are direct measurements, uncorrected for drying shrinkage. Statistical tests are the equal variance F-test and the equal mean t-test (t_t_). The critical P-value of <0.05 was used to signify difference, i. e. the symbol < if the left mean is significantly lower than the right mean, the symbol > if the left mean is significantly higher than the right mean and the symbol = if the difference between the two means is insignificant. Relative difference between means is shown as a percentage (%) of the difference between the means divided by the mean of the lower value, a negative sign indicates that the left mean is lower than the right mean.

| Region |  | 2014 |  |  |  |  | 2017 |  |  |
| --- | --- | --- | --- | --- | --- | --- | --- | --- | --- |
|  | Size, μm | Mean | SD | n | t_t_ | % | Mean | SD | n |
| AtlO | Length | 1.30 | 0.24 | 25 | = | 5.6 | 1.23 | 0.13 | 7 |
|  | Width | 1.13 | 0.22 | 25 | = | 2.4 | 1.10 | 0.17 | 7 |
|  | Tinsel | 3.55 | 0.46 | 22 | = | 9.0 | 3.26 | 0.51 | 6 |
|  | Caudal | 1.37 | 0.26 | 21 | > | 41.3 | 0.97 | 0.15 | 3 |
|  | Whip | 0.72 | 0.09 | 15 | = | -1.6 | 0.73 | 0.20 | 3 |
| AtlM | Length | 1.52 | 0.26 | 30 | = | 6.6 | 1.42 | 0.27 | 15 |
|  | Width | 1.27 | 0.24 | 30 | > | 14.3 | 1.12 | 0.22 | 15 |
|  | Tinsel | 3.53 | 0.35 | 26 | > | 9.0 | 3.24 | 0.27 | 15 |
|  | Caudal | 1.19 | 0.23 | 23 | = | 1.5 | 1.18 | 0.13 | 13 |
|  | Whip | 0.65 | 0.12 | 16 | = | 4.6 | 0.62 | 0.11 | 4 |

Next, we compared dimensions of the four HK morphotypes in the AtlO and SPacO (**Supplementary Table 9**). Of all dimensions compared only the width of mcHK cells showed significant difference but of merely 18.5%, which could reflect the difference in the mcHK growth. Because all the other dimensions were similar, we could conclude that each one of the four HK morphotypes is indistinguishable between the two oligotrophic oceans.

**Supplementary Table 9.** Comparison of cell (length and width) and flagella (tinsel, caudal and whip) sizes of the short tinsel heterokont (stHK), short caudal heterokont (scHK), medium caudal heterokont (mcHK) and long caudal heterokont (lcHK) morphotypes in the Atlantic and South Pacific oligotrophic (AtlO and SPacO) waters. Cell dimensions are direct measurements, uncorrected for drying shrinkage. Statistical tests are the equal variance F-test and the equal mean t-test (t_t_). The critical P-value of <0.05 was used to signify difference, i. e. the symbol < if the left mean is significantly lower than the right mean, the symbol > if the left mean is significantly higher than the right mean and the symbol = if the difference between the two means is insignificant. Relative difference between means is shown as a percentage (%) of the difference between the means divided by the mean of the lower value, a negative sign indicates that the left mean is lower than the right mean.

| Morphotype |  | AtlO |  |  |  |  | SPacO |  |  |
| --- | --- | --- | --- | --- | --- | --- | --- | --- | --- |
|  | Size, μm | Mean | SD | n | t_t_ | % | Mean | SD | n |
| stHK | Length | 1.29 | 0.22 | 32 | = | -2.9 | 1.32 | 0.22 | 97 |
|  | Width | 1.12 | 0.21 | 32 | = | -0.8 | 1.13 | 0.19 | 97 |
|  | Tinsel | 3.49 | 0.48 | 28 | = | -4.7 | 3.65 | 0.38 | 94 |
|  | Caudal | 1.32 | 0.28 | 24 | = | 0.6 | 1.31 | 0.27 | 82 |
|  | Whip | 0.72 | 0.10 | 18 | = | 3.8 | 0.70 | 0.15 | 63 |
| scHK | Length | 1.39 | 0.24 | 10 | = | -9.2 | 1.51 | 0.17 | 4 |
|  | Width | 1.07 | 0.14 | 10 | = | -12.0 | 1.20 | 0.16 | 4 |
|  | Tinsel | 8.67 | 0.91 | 6 | = | -0.2 | 8.69 | 1.23 | 4 |
|  | Caudal | 0.71 | 0.19 | 7 | = | -18.6 | 0.85 | 0.11 | 3 |
|  | Whip | 1.84 | 0.64 | 5 | = | 33.9 | 1.37 | 0.54 | 3 |
| mcHK | Length | 1.54 | 0.27 | 8 | = | -9.3 | 1.68 | 0.22 | 9 |
|  | Width | 1.10 | 0.20 | 8 | < | -18.5 | 1.31 | 0.15 | 9 |
|  | Tinsel | 9.05 | 1.90 | 8 | = | 0.4 | 9.01 | 1.95 | 9 |
|  | Caudal | 1.47 | 0.35 | 7 | = | -13.8 | 1.67 | 0.18 | 5 |
|  | Whip | 1.57 | 0.29 | 4 | = | 1.7 | 1.54 | 0.43 | 4 |
| lcHK | Length | 2.03 | 0.52 | 9 | = | 8.7 | 1.87 | 0.30 | 10 |
|  | Width | 1.65 | 0.50 | 9 | = | 4.8 | 1.57 | 0.20 | 10 |
|  | Tinsel | 10.0 | 0.80 | 6 | = | -2.8 | 10.3 | 2.11 | 10 |
|  | Caudal | 3.04 | 0.62 | 9 | = | 2.2 | 2.97 | 0.37 | 10 |
|  | Whip | 1.70 | 0.88 | 7 | = | 14.6 | 1.48 | 0.35 | 4 |

Comparison of the four HK morphotypes in the AtlM and SPacM waters (**Supplementary Table 10**) showed their general similarity although at least one of the dimensions of each morphotype was statistically different. The tinsel and caudal flagella of the stHK were 6.2% and 16.2% longer in the AtlM. The cells of the scHK were 22.3% longer in the AtlM. The caudal flagella of the mcHK were 44% shorter in the AtlM. The cells of the lcHK were 28.7% narrower in the AtlM. The differences of the scHK, mcHK and lcHK could owe their significance to a limited number (n<10) of the analysed specimens in the AtlM. The differences of the stHK although significant are relatively minor (<20%) and hence considered insufficient to rule out similarity of the stHK in the AtlM and SPacM.

**Supplementary Table 10.** Comparison of cell (length and width) and flagella (tinsel, caudal and whip) sizes of the short tinsel heterokont (stHK), short caudal heterokont (scHK), medium caudal heterokont (mcHK) and long caudal heterokont (lcHK) morphotypes in the Atlantic and South Pacific mesotrophic regions (AtlM and SPacM, respectively). Cell dimensions are direct measurements, uncorrected for drying shrinkage. Statistical tests are the equal variance F-test and the equal mean t-test (t_t_). The critical P-value of <0.05 was used to signify difference, i. e. the symbol < if the left mean is significantly lower than the right mean, the symbol > if the left mean is significantly higher than the right mean and the symbol = if the difference between the two means is insignificant. Relative difference between means is shown as a percentage (%) of the difference between the means divided by the mean of the lower value, a negative sign indicates that the left mean is lower than the right mean.

| Morphotype |  | AtlM |  |  |  |  | SPacM |  |  |
| --- | --- | --- | --- | --- | --- | --- | --- | --- | --- |
|  | Size, μm | Mean | SD | n | t_t_ | % | Mean | SD | n |
| stHK | Length | 1.41 | 0.29 | 62 | = | 1.7 | 1.38 | 0.25 | 94 |
|  | Width | 1.14 | 0.26 | 94 | = | -2.5 | 1.17 | 0.20 | 94 |
|  | Tinsel | 3.40 | 0.34 | 51 | > | 6.2 | 3.20 | 0.40 | 68 |
|  | Caudal | 1.20 | 0.19 | 42 | > | 16.2 | 1.03 | 0.19 | 62 |
|  | Whip | 0.65 | 0.11 | 26 | = | 3.7 | 0.63 | 0.14 | 46 |
| scHK | Length | 1.70 | 0.35 | 8 | > | 22.3 | 1.39 | 0.19 | 58 |
|  | Width | 1.22 | 0.27 | 8 | = | 11.5 | 1.10 | 0.16 | 58 |
|  | Tinsel | 8.37 | 0.98 | 8 | = | 3.4 | 8.09 | 1.10 | 58 |
|  | Caudal | ND |  |  |  |  | 0.69 | 0.23 | 27 |
|  | Whip | 0.82 | ND | 1 |  |  | 1.27 | 0.46 | 27 |
| mcHK | Length | 1.71 | 0.37 | 12 | = | -9.1 | 1.87 | 0.38 | 56 |
|  | Width | 1.26 | 0.36 | 12 | = | -11.2 | 1.40 | 0.22 | 56 |
|  | Tinsel | 9.49 | 2.12 | 10 | = | 10.1 | 8.62 | 1.49 | 32 |
|  | Caudal | 1.38 | 0.22 | 8 | < | -44.1 | 1.99 | 0.46 | 30 |
|  | Whip | 1.08 | 0.79 | 6 | = | -29.1 | 1.39 | 0.40 | 22 |
| lcHK | Length | 1.80 | 0.46 | 9 | = | -8.8 | 1.96 | 0.20 | 24 |
|  | Width | 1.22 | 0.21 | 9 | < | -28.7 | 1.57 | 0.20 | 24 |
|  | Tinsel | 9.96 | 1.27 | 5 | = | 5.6 | 9.43 | 1.89 | 14 |
|  | Caudal | 3.18 | 0.58 | 9 | = | -13.2 | 3.60 | 0.77 | 19 |
|  | Whip | 1.46 | 0.48 | 7 | = | 21.6 | 1.20 | 0.73 | 7 |

ND, not determined.

Assuming general similarity of the four HK morphotypes in the Atlantic and Pacific Oceans, we pooled the data to compare the morphotypes in the oligotrophic and mesotrophic waters (**Supplementary Table 11**). The consequential considerable increase of independent measurements made the t-test more stringent, however, none of the differences exceeded the threshold 20% except the whip length of the scHK (n<10). Hence, each one of the four HK morphotypes is statistically indistinguishable between the oligotrophic and mesotrophic waters and the data collected could be pooled together to assess mean values for the entire open ocean (**Supplementary Table 6**).

**Supplementary Table 11.** Comparison of cell (length and width) and flagella (tinsel, caudal and whip) sizes of the short tinsel heterokont (stHK), short caudal heterokont (scHK), medium caudal heterokont (mcHK) and long caudal heterokont (lcHK) morphotypes in the Atlantic and South Pacific oligotrophic waters versus the Atlantic and South Pacific mesotrophic waters. Statistical tests are the equal variance F-test and the equal mean t-test (t_t_). The critical P-value of <0.05 was used to signify difference, i. e. the symbol < if the left mean is significantly lower than the right mean, the symbol > if the left mean is significantly higher than the right mean and the symbol = if the difference between the two means is insignificant. Relative difference between means is shown as a percentage (%) of the difference between the means divided by the mean of the lower value, a negative sign indicates that the left mean is lower than the right mean.

| Morphotype |  | Oligotrophic | |  |  |  | Mesotrophic | |  |
| --- | --- | --- | --- | --- | --- | --- | --- | --- | --- |
|  | Size, μm | Mean | SD | n | t_t_ | % | Mean | SD | n |
| stHK | Length | 1.31 | 0.22 | 129 | < | -6.0 | 1.39 | 0.27 | 156 |
|  | Width | 1.13 | 0.20 | 129 | = | -2.7 | 1.16 | 0.23 | 156 |
|  | Tinsel | 3.62 | 0.41 | 122 | > | 10.1 | 3.28 | 0.39 | 119 |
|  | Caudal | 1.31 | 0.27 | 106 | > | 19.2 | 1.10 | 0.21 | 104 |
|  | Whip | 0.70 | 0.14 | 81 | > | 10.9 | 0.63 | 0.13 | 72 |
| scHK | Length | 1.42 | 0.22 | 14 | = | -0.2 | 1.42 | 0.24 | 66 |
|  | Width | 1.11 | 0.15 | 14 | = | -0.1 | 1.11 | 0.18 | 66 |
|  | Tinsel | 8.68 | 0.98 | 10 | = | 6.8 | 8.13 | 1.09 | 66 |
|  | Caudal | 0.75 | 0.17 | 10 | = | 9.3 | 0.69 | 0.23 | 27 |
|  | Whip | 1.66 | 0.61 | 8 | > | 32.9 | 1.25 | 0.46 | 28 |
| mcHK | Length | 1.61 | 0.25 | 17 | < | -13.9 | 1.84 | 0.38 | 68 |
|  | Width | 1.21 | 0.20 | 17 | < | -13.5 | 1.37 | 0.25 | 68 |
|  | Tinsel | 9.03 | 1.87 | 17 | = | 2.3 | 8.83 | 1.67 | 42 |
|  | Caudal | 1.55 | 0.30 | 12 | < | -19.6 | 1.86 | 0.49 | 38 |
|  | Whip | 1.55 | 0.34 | 8 | = | 17.6 | 1.32 | 0.51 | 28 |
| lcHK | Length | 1.95 | 0.41 | 19 | = | 1.8 | 1.91 | 0.29 | 33 |
|  | Width | 1.61 | 0.36 | 19 | = | 8.9 | 1.47 | 0.25 | 33 |
|  | Tinsel | 10.20 | 1.71 | 16 | = | 6.6 | 9.57 | 1.73 | 19 |
|  | Caudal | 3.00 | 0.49 | 19 | < | -15.3 | 3.46 | 0.73 | 28 |
|  | Whip | 1.62 | 0.72 | 11 | = | 21.5 | 1.32 | 0.61 | 14 |

**Supplementary Table 12.** Comparison of relative standard deviations (R-SD) of the cell (length and width) and flagella (tinsel, non-tinsel or “caudal” and its whip) dimensions of the choanoflagellate (Cho) representative species, *Coronoeca conicella* (*C.c.*), *Stephanacantha oceanica* (*S.o.*), average loricate and aloricate choanoflagellates (lCho and aCho, respectively) as well as long caudal heterokont (lcHK), the medium caudal heterokont (mcHK), the short caudal heterokont (scHK) and the short tinsel heterokont (stHK) morphotypes in the open ocean plus short tinsel heterokont morphotype at the station SPacO_3 (stHK^#^) in the South Pacific oligotrophic waters and three isolates of *Pteridomonas danica* with small, medium and large cells (*P.d.* S, *P.d.* M and *P.d.* L, respectively). Variances of relative measurements as fractions of means were compared using Fisher’s F-test (F_t_) with critical P-value of <0.05 to signify difference. The R-SD is the square root of the relative variances. The symbols >, < or = indicate that the left R-SD is significantly higher, lower or insignificantly different from the right R-SD, respectively. Note the R-SDs of the width and non-tinsel flagellum were compared for Cho.

| Morphotype | | Length | F_t_ | Width | | F_t_ | Tinsel | | F_t_ | Non-tinsel | | F_t_ | Whip | |
| --- | --- | --- | --- | --- | --- | --- | --- | --- | --- | --- | --- | --- | --- | --- |
|  |  | R-SD |  | R-SD | n |  | R-SD | n |  | R-SD | n |  | R-SD | n |
| Cho | *C.c.* | 0.171 | = | 0.133 | 14 | = |  |  |  | 0.192 | 9 | = | 0.382 | 5 |
|  | *S.o.* | 0.209 | = | 0.193 | 21 | = |  |  |  | 0.177 | 16 | = | 0.333 | 3 |
|  | lCho | 0.299 | > | 0.214 | 132 | < |  |  |  | 0.341 | 64 | < | 0.602 | 23 |
|  | aCho | 0.162 | = | 0.147 | 46 | = |  |  |  | 0.155 | 37 | < | 0.483 | 22 |
| HK | lcHK | 0.175 | = | 0.191 | 52 | = | 0.172 | 35 | = | 0.191 | 47 | < | 0.441 | 25 |
|  | mcHK | 0.197 | = | 0.181 | 85 | = | 0.193 | 59 | < | 0.246 | 50 | < | 0.350 | 36 |
|  | scHK | 0.164 | = | 0.155 | 80 | = | 0.131 | 76 | < | 0.311 | 37 | = | 0.364 | 36 |
|  | stHK | 0.180 | = | 0.186 | 285 | > | 0.115 | 241 | < | 0.200 | 209 | = | 0.200 | 153 |
|  | stHK^#^ | 0.151 | = | 0.153 | 78 | > | 0.096 | 77 | < | 0.165 | 67 | < | 0.221 | 50 |
| *P.d.* | S | 0.093 | = | 0.093 | 28 | = | 0.096 | 27 |  |  |  |  |  |  |
|  | M | 0.097 | = | 0.092 | 58 | = | 0.099 | 56 |  |  |  |  |  |  |
|  | L | 0.081 | = | 0.086 | 24 | < | 0.138 | 22 |  |  |  |  |  |  |

**Supplementary Video**
